# Gut-associated functions are favored during microbiome assembly across a major part of *C. elegans* life

**DOI:** 10.1128/mbio.00012-24

**Published:** 2024-04-18

**Authors:** Johannes Zimmermann, Agnes Piecyk, Michael Sieber, Carola Petersen, Julia Johnke, Lucas Moitinho-Silva, Sven Künzel, Lena Bluhm, Arne Traulsen, Christoph Kaleta, Hinrich Schulenburg

**Affiliations:** 1 Research Group Evolutionary Ecology and Genetics, Zoological Institute, Kiel University, Kiel, Germany; 2 Max Planck Fellow Group Antibiotic Resistance Evolution, Max Planck Institute for Evolutionary Biology, Ploen, Germany; 3 Research Group Medical Systems Biology, Institute of Experimental Medicine, Kiel University, Kiel, Germany; 4 Department for Evolutionary Theory, Max Planck Institute for Evolutionary Biology, Ploen, Germany; ^5^Institute of Clinical Molecular Biology, Christian-Albrechts University, Kiel, Germany; 6 Department of Evolutionary Genetics, Max Planck Institute for Evolutionary Biology, Ploen, Germany; Harvard University, Cambridge, Massachusetts, USA

**Keywords:** microbiome, systems biology, microbial communities, microbial ecology, *Caenorhabditis elegans*, community dynamics, functional ecology

## Abstract

The microbiome expresses a variety of functions that influence host biology. The range of functions depends on the microbiome’s composition, which can change during the host’s lifetime due to neutral assembly processes, host-mediated selection, and environmental conditions. To date, the exact dynamics of microbiome assembly, the underlying determinants, and the effects on host-associated functions remain poorly understood. Here, we used the nematode *Caenorhabditis elegans* and a defined community of fully sequenced, naturally associated bacteria to study microbiome dynamics and functions across a major part of the worm’s lifetime of hosts under controlled experimental conditions. Bacterial community composition initially shows strongly declining levels of stochasticity, which increases during later time points, suggesting selective effects in younger animals as opposed to more random processes in older animals. The adult microbiome is enriched in genera *Ochrobactrum* and *Enterobacter* compared to the direct substrate and a host-free control environment. Using pathway analysis, metabolic, and ecological modeling, we further find that the lifetime assembly dynamics increase competitive strategies and gut-associated functions in the host-associated microbiome, indicating that the colonizing bacteria benefit the worm. Overall, our study introduces a framework for studying microbiome assembly dynamics based on stochastic, ecological, and metabolic models, yielding new insights into the processes that determine host-associated microbiome composition and function.

**IMPORTANCE:**

The microbiome plays a crucial role in host biology. Its functions depend on the microbiome composition that can change during a host’s lifetime. To date, the dynamics of microbiome assembly and the resulting functions still need to be better understood. This study introduces a new approach to characterize the functional consequences of microbiome assembly by modeling both the relevance of stochastic processes and metabolic characteristics of microbial community changes. The approach was applied to experimental time-series data obtained for the microbiome of the nematode *Caenorhabditis elegans* across the major part of its lifetime. Stochastic processes played a minor role, whereas beneficial bacteria as well as gut-associated functions enriched in hosts. This indicates that the host might actively shape the composition of its microbiome. Overall, this study provides a framework for studying microbiome assembly dynamics and yields new insights into *C. elegans* microbiome functions.

## INTRODUCTION

Microorganisms have shaped the evolution of multicellular host organisms since the very beginning (1). As a consequence, they are of particular importance for the biology of their hosts. They can influence host nutrition and metabolism by providing specific enzymes for digestion (e.g., cellulase for digestion of plant material [2]) or certain nutritious compounds (e.g., specific amino acids [3]). They can define host development (4), maturation of the immune system (5), mediate immune protection against pathogens (6), or even influence host behavior through interaction with the host’s nervous system (7). During the lifetime of a host, the composition of its associated microbiome can change. For example, the assembly of the zebrafish gut microbiome is initially driven by neutral processes, while later time points during development are characterized by non-neutral dynamics, most likely due to maturation of the immune system (8). Overall, microbiome changes can be described by Vellend’s four high-level processes: selection, dispersal, drift, and speciation/diversification (9). The four processes help to generalize specific causes underlying microbial assembly, such as priority effects (selection), neutral assembly (drift, dispersal, and speciation), selection by the host, and interaction of microbes with the host or with other microbes (selection) (9, 10). To date, however, it is still poorly understood how these temporal changes affect the microbiome-mediated functions. If the host controls the microbiome assembly process, then functional changes may be expected because the host usually faces different functional challenges during its lifetime (e.g., development and immune protection early in life, sexual reproduction after maturation, etc.). If the bacteria are in control, then later time points should be characterized by bacteria well adapted to the host habitat and/or highly competitive bacteria. A controlled experimental approach may help to better understand the consequences of microbiota assembly changes during host lifetime.

The nematode *Caenorhabditis elegans* provides a tractable experimental system, for which a large number of associated microorganisms have been isolated together with the nematode from the host’s natural habitat: rotting plant matter (11, 12). Naturally associated microorganisms include members of the genera *Pseudomonas*, *Ochrobactrum*, *Enterobacter*, *Comamonas,* and others. Previous work highlighted that a representative set of the naturally associated microbes can provide almost all essential nutrients needed by the worm for growth (except cholesterol) and significantly affect *C. elegans* population growth (13). Naturally associated bacteria can moreover enrich in worms and provide immune protection against pathogens such as the gram-negative *Bacillus thuringiensis*, the gram-positive *Pseudomonas aeruginosa*, or the fungus *Drechmeria coniospora* (11, 14–16).

Since changes of microbial composition are dynamic and at least partially dependent on host characteristics during development and adult life, we combined microbial abundance data from the different sampling time points with results from ecological and metabolic modeling to study consequences of these changes at the functional level. Microbiome compositional changes can be studied across time using neutral assembly models, and metabolic network models can be employed to uncover functions. In particular, we found surprisingly little compatibility with a neutral model for the microbial assembly in a former study which we intended to follow on longer time scales (17). In addition, we recently developed a new method for metabolic pathway and model prediction (18) and proposed a classification of ecological strategies for microbes (13).

The aim of the current study was to use *C. elegans* and 43 bacteria representative of the nematode’s naturally associated microbiome (i.e., CeMbio43) in order to assess microbiome assembly dynamics and resulting functional consequences during the major part of the lifetime of the host. These 43 bacteria are an extension of the set of 12 bacteria that define the previously published CeMbio resource (19). We obtained full-genome sequences and reconstructed metabolic network models for these 43 bacteria. The bacteria were either combined with *C. elegans* or not (as control). Microbiome composition was characterized with the help of 16S rDNA amplicon sequencing for nematodes and the directly associated environment (labeled “substrate”) or the control environments without worms (labeled “control”) at six time points, covering *C. elegans* development and a considerable part of its adult lifetime (almost 8 days after egg hatching at 25°C). We assessed diversity and neutrality of microbiome dynamics. We further used the reconstructed metabolic networks to infer changes in the metabolic competences of the microbial communities.

## MATERIALS AND METHODS

An extended version of material and methods can be found in the supplemental material.

### Material

We used the *C. elegans* strain DH26, which has a mutation in the gene *rrf-3*, encoding a member of the RNA-dependent RNA polymerase family. As possible for any mutation in a central gene, the *rrf-3* variant may affect microbiome assembly (20). Nevertheless, this mutant comes with the unique advantage that it is spermatogenesis defective and sterile at 25°C. Therefore, using this mutant allows us to avoid overlapping generations in our experiment, thereby enabling us to sample repeatedly across the lifetime of this nematode. We defined a community of 43 natural *C. elegans* microbiome isolates, the CeMbio43 (Table 1; Tables S1 and S2), which extends the previously described CeMbio resource (consisting of 12 bacterial strains [19]), which is representative of the natural *C. elegans* microbiome (11, 12), and which includes several strains for the naturally more abundant genera in the worm’s microbiome.

**TABLE 1:** Overview of the extended CeMbio community including genome statistics^*a*^

ID	Species	Ref^*b*^	Mb^*c*^	Genes	Contigs	rRNA
BIGb0170	*Sphingobacterium multivorum*	*	6.4	5,391	1	21
BIGb0172	*Comamonas piscis*	*	5.2	4,595	1	19
BIGb0393	*Pantoea nemavictus*	*	5.2	4,667	2	22
CEent1	*Enterobacter cloacae*	*	4.8	4,458	1	26
JUb134	*Sphingomonas molluscorum*	*	4.1	3,816	4	9
JUb19	*Stenotrophomonas indicatrix*	*	4.6	4,079	2	14
JUb44	*Chryseobacterium scophthalmum*	*	4.7	4,199	1	21
JUb66	*Lelliottia amnigena*	*	4.6	4,207	1	22
MSPm1	*Pseudomonas berkeleyensis*	*	5.7	5,159	2	12
MYb10	*Acinetobacter guillouiae*	*	4.6	4,244	29	3
MYb11	*Pseudomonas lurida*	*	6.1	5,456	1	16
MYb115	*Pseudomonas fluorescens*	(**)	6.3	5,676	1	22
MYb121	*Erwinia billingiae*	**	5.0	4,532	28	6
MYb158	*Acinetobacter johnsonii*		3.6	3,414	23	3
MYb174	*Enterobacter ludwigii*		4.6	4,293	29	5
MYb176	*Enterobacter ludwigii*		4.6	4,283	37	3
MYb177	*Acinetobacter* sp. B2070		3.5	3,311	2	21
MYb181	*Sphingobacterium faecium*	**	5.5	4,619	243	8
MYb186	*Enterobacter* sp. 638		4.8	4,485	26	3
MYb191	*Acinetobacter* sp. 4D-W-22		3.5	3,350	21	11
MYb21	*Comamonas* sp. B-9		5.4	4,762	36	4
MYb25	*Chryseobacterium culicis*	**	5.0	4,504	11	3
MYb264	*Chryseobacterium* sp.		5.2	4,607	1	21
MYb328	*Chryseobacterium* sp.		5.6	5,004	60	3
MYb330	*Pseudomonas* sp.		6.1	5,446	65	4
MYb331	*Pseudomonas* sp.		6.1	5,447	71	4
MYb371	*Pseudomonas* sp.		6.1	5,450	53	4
MYb375	*Erwinia* sp.		4.9	4,505	24	11
MYb379	*Ochrobactrum* sp.		5.1	4,780	47	5
MYb382	*Sphingobacterium* sp.		4.7	4,002	29	11
MYb388	*Sphingobacterium* sp.		6.2	5,326	29	3
MYb396	*Comamonas* sp.		5.3	4,719	23	3
MYb398	*Pseudomonas* sp.		5.5	4,895	105	4
MYb416	*Erwinia* sp.		5.8	5,380	443	14
MYb49	*Ochrobactrum anthropi*	**	4.8	4,553	3	12
MYb535	*Erwinia* sp.		4.9	4,599	25	11
MYb541	*Pseudomonas* sp.		6.3	5,789	82	4
MYb58	*Ochrobactrum pituitosum*	**	5.0	4,635	3	15
MYb592	*Gluconobacter wancherniae*		3.2	3,048	40	6
MYb595	*Gluconobacter cerinus*		3.6	3,299	48	2
MYb596	*Gluconobacter albidus*		3.3	3,044	51	3
MYb69	*Comamonas* sp. TK41		5.3	4,610	28	3
MYb71	*Ochrobactrum vermis*	*	5.4	5,191	3	12

^*a*^ Illumina short reads were used from reference 13, and additional Pacbio long reads were obtained for this study.

^*b*^ Reference studies which previously described the respective genomes: “*” Dirksen et al. (19) and “**” Zimmermann et al. (13).

^*c*^ Genome size given in mega base pairs (Mb).

### Whole-genome sequence analysis of bacteria

We isolated high-quality genomic DNA using the Nucleo Spin Tissue Kit (Macherey-Nagel, Düren, Germany) for the 26 bacterial strains without previously sequenced genomes (Table 1). Short-read sequencing was performed by NextSeq 500 using the NextSeq 500/550 High Output Kit v2.5 (300 cycles) and long-read sequencing with the Pacbio Sequel II. The genomes were assembled using SPAdes v3.14.1 (21), MaSuRCA 3.4.2 (22), Unicycler v0.4.8 (23), shovill 1.1.0 (24), and SKESA 2.4.0 (25). Genome assembly quality was evaluated using QUAST v5.1.0rc1 (26). The genome-sequencing data were used to annotate genomic functions with gapseq 1.1 (Sequence DB: 1139b8e, bitscore cutoff 150 [18]), including inference of metabolic pathways (27), metabolic network models, growth media suitability, and carbon source and fermentation products. The variation in metabolic reactions between the CeMbio43 strains predicted by gapseq was analyzed by a multiple correspondence analysis (MCA) based on the presence or absence of reactions in metabolic models using the function *MCA*() of the R package FactoMineR v2.9 using strains as individuals and reactions as categorical variables (28). Virulence, resistance genes, and plasmids were scanned with abricate 1.0.1 (29) using the databases vfdb 2018-Jul-16 (30), plasmidfinder 2022-Mar-8 (31), resfinder 2022-Mar-8 (32), and megares 2022-Mar-8 (33). Gut microbiome-specific gene clusters were identified by gutSMASH 1.0.0-1555cd7 (34). For CAzyme annotation, dbCAN 3.0.2 was used (35). The microbial interactions were simulated by a pairwise comparison of single vs co-growth rate assuming TSB growth medium with BacArena 1.8.2 (36).

### Analysis of microbiome changes

Temporal changes in microbiota composition were assessed for nematode populations (labeled “host”), the directly connected nematode growth medium (NGM) environment (labeled “substrate”), and the control NGM plates without worms (labeled “control”) in 8–13 replicates and across 6 time points. The experiment was started with L1 larvae of *C. elegans* strain DH26. Nematodes and bacterial lawns were sampled after 16 h (*t*2), 42 h (*t*3), 66 h (*t*4), 90 h (*t*5), 138 h (*t*6), and 186 h (*t*7). DNA was extracted with the Nucleo Spin 96 Tissue Kit (Macherey-Nagel, Düren, Germany). The 16S libraries were prepared using the 341F and 806R primer covering the V3-V4 region of the 16S rRNA gene, followed by sequencing on the Miseq platform (Illumina). We processed the obtained 16S amplicon-sequencing data using the standardized amplicon-sequencing pipeline nf-core/ampliseq 2.1.1 (37). Sample sequences were inferred by DADA2 (38), and the microbial composition data (ASV and taxonomic table) were analyzed with phyloseq 1.38.0 (39). DESeq2 1.34.0 was used to find differentially abundant taxa (40) that were visualized by heat trees using the R package metacoder 0.3.5.001 (41). To assess the neutrality of the samples, the expected long-term distribution of a neutral model was fitted to the sample abundance data (17, 42). The impact of stochastic processes on community assembly was evaluated using the normalized stochasticity ratio metric available as R package NST 3.1.10 (function tNST [43]). We combined the microbial 16S data with the functions predicted from genomic analysis and modeling. For each organism, the presence (true/false) of a particular function indicated if the organism’s abundance contributed to the abundance of the function. Finally, by adding up the abundances of all taxa in a sample for which the presence of a function was predicted, the overall abundance of the function was determined. In analogy to the taxon-level analysis, DESeq2 1.34.0 was used to identify differentially abundant functions.

## RESULTS

### The extended CeMbio43 community, genomes, and metabolic models

**Fig 1:**
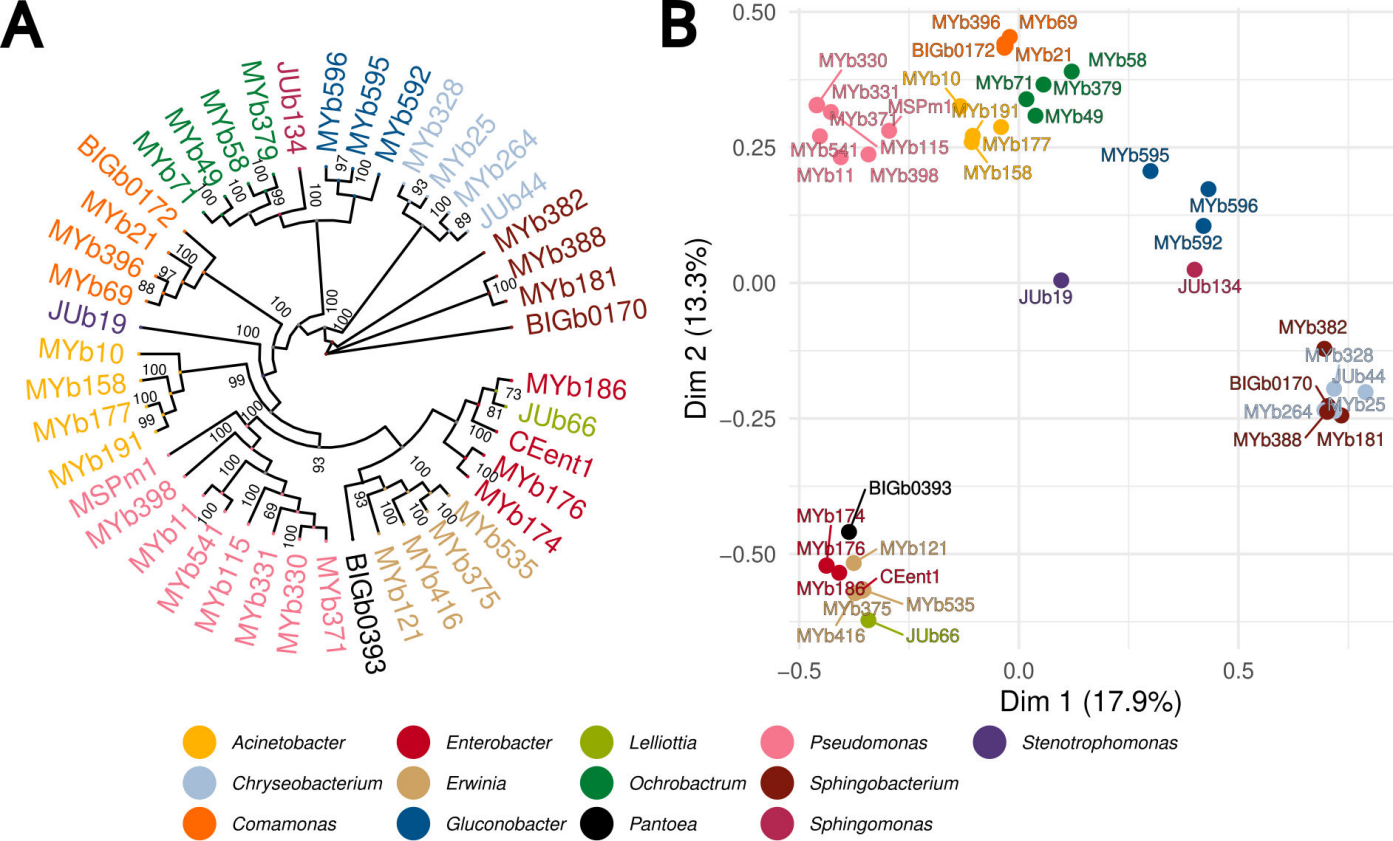
Genomes and inferred metabolic model similarities: (**A**) Rooted phylogenetic tree in circular format based on assembled genome similarity (alignment by GtdbTk [45] and tree by IQ-TREE [46], midpoint rooting), (**B**) Multiple correspondence analysis of reactions that were present in the inferred metabolic models. The colors indicate the different genera of the CeMbio43 community. Strain codes are as in Table 1.

In 2020, we introduced a natural *Caenorhabditis elegans* microbiome resource, CeMbio, consisting of 12 representative members of the nematode’s native microbiome (19). In the current study, we present an extended version of the CeMbio community with 43 bacterial strains, CeMbio43, which incorporates additional taxa chosen by their repeated presence in a long-term field study (11, 12). Newly integrated strains belong to the genera *Erwinia*, *Microbacterium*, and *Gluconobacter*. Besides a more comprehensive coverage of representative taxa of the native microbiome, the CeMbio43 community accounts for the redundancy detected for many microbiomes (44). Additional strains to increase redundancy were added for *Acinetobacter* (+3), *Chryseobacterium* (+3), *Comamonas* (+3), *Enterobacter* (+3), *Ochrobactrum* (+3), *Sphingobacterium* (+3), and *Pseudomonas* (+6). Overall, the CeMbio43 community augments the existing resources for microbiome research in *C. elegans* by further improving the representation of a native microbiome and by accounting for species redundancy (Table 1; Tables S1 and S2; Fig. 1).

For the 43 strains of the community, 12 genomes were already assembled for the original CeMbio community (19), and an additional 5 were already characterized for a previous study (13). We now assembled the genomes for the remaining 26 strains. Noteworthy, depending on the organisms, the best assemblies were not obtained from one single but a combination of methods (MaSuRCA, shovill, SPAdes, and Unicycler). SPAdes made more than one-third of the high-performing assemblies (Table S2). The CeMbio43 genomes were used to reconstruct a phylogenetic tree and, importantly, infer metabolic models and thus the metabolic competences of the included strains (Fig. 1).

### Microbial community changes across the major part of nematode life

**Fig 2:**
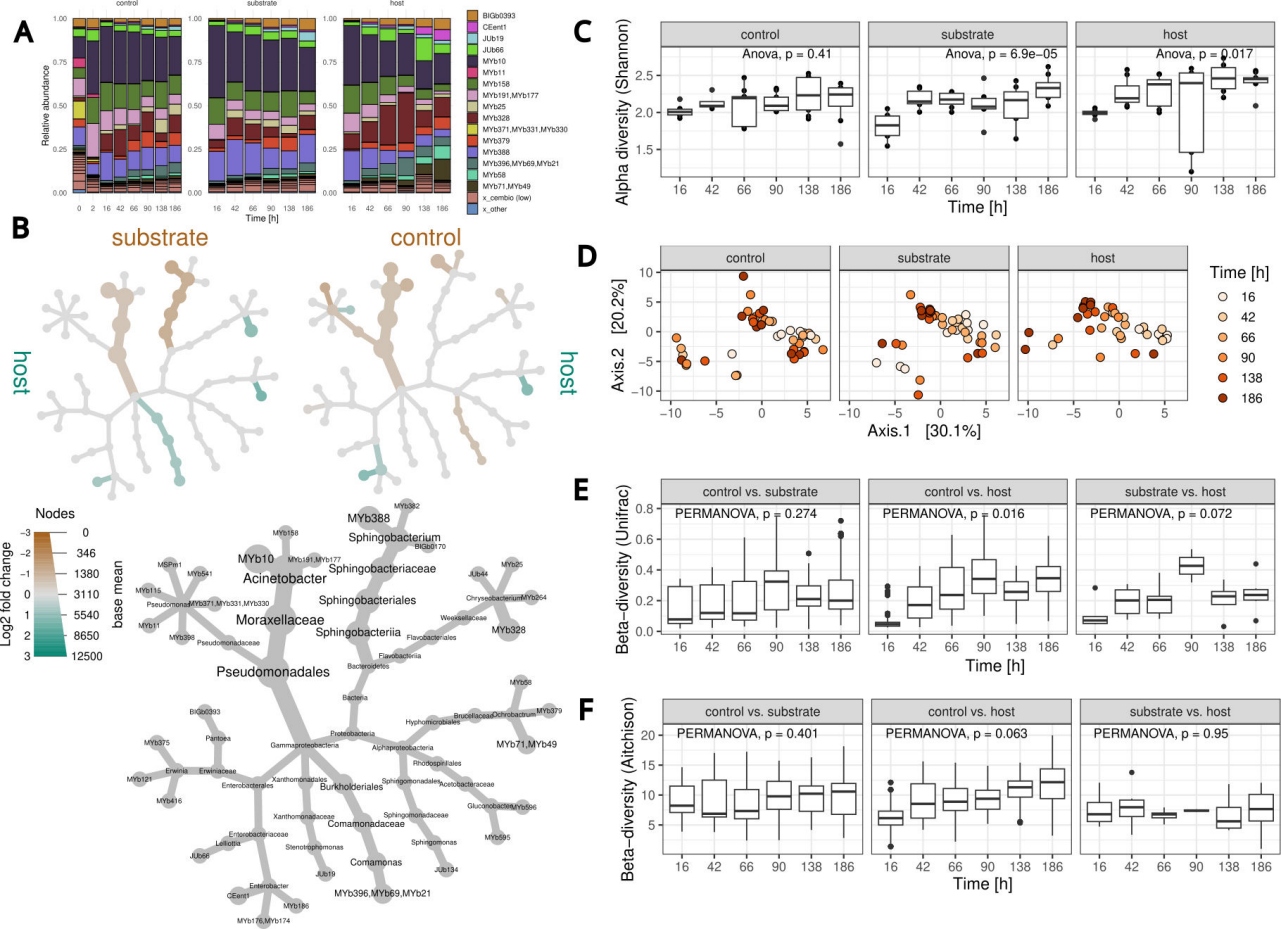
Microbial abundances and diversity across sample types and time. (**A**) Relative mean abundances per sample type and time point. Only taxa with a maximal relative abundance ≥0.05 are shown. Low abundance CeMbio43 taxa [group: “cembio (low)”] and unidentified taxa (group: “other”) are represented jointly. (**B**) Differential heat tree of species abundances. A taxonomic tree is shown in gray. Differentially abundant species between three conditions were compared: microbial samples from *C. elegans* (host), from the plate environment directly associated with the worms (substrate), and from plate environments that were always maintained without worms (controls). The colored trees indicate the comparison: substrate vs host (top left), control vs host (top right). Differentially abundant species were colored brown (enriched in substrate or controls) or turquoise (enriched in hosts). Differential abundances were inferred by DESeq2. Taxa with adjusted *P*-value of ≤0.05 are shown. The thickness of branches corresponds to mean abundance. (**C**) *α*-Diversity of filtered and agglomerated microbial samples on taxon level (Shannon diversity) across time. (**D**) Ordination of microbial samples shown in a PCoA plot using Aitchison distance. Time is indicated by increasing red color intensities. (**E** and **F**) Differences in *β*-diversity (Aitchison, weighted Unifrac distance) compared between sample types and across time. For comparing host-associated samples, pairs from the same replicate were available, whereas for comparing host with control or substrate with control, pairs were randomly associated (100 repetitions).

We studied changes in microbial communities from three environmental sources (control, substrate, and host) for six time points (16, 42, 66, 90, 138, 186 h) with 8–13 replicates, totaling 180 samples. The experiment was started with synchronized L1 larvae (time point *t*0), whereby the first two (16, 42 h) cover larval development, while the remaining four time points encompass a large part of adult life, including the usual reproductive period of *C. elegans* at this temperature. Microbial community composition was characterized for nematode hosts, the plate environment from which nematodes were isolated (labeled substrate), and also as controls, plate environments maintained without *C. elegans*. We measured the within-sample diversity and compared the influence of time and sample source on community dynamics, illustrated for the considered genera in the barplot of Fig. 2A. The diversity increase over time was most robust for host and substrate samples (Spearman *R* = 0.52; cf. *R* = 0.32 [controls]; Fig. S5). The Shannon index showed significant temporal variation for substrate and host samples (Fig. 2C). While a similar result was obtained for evenness (Simpson), richness (Chao1) did not vary significantly over time (Fig. S6). Analysis of the variance (ANOVA) of Shannon indices indicated the influence of sample source and time (Table S5). The Tukey honest significant difference test suggested significant differences at the beginning and between 90 and 186 h (Table S6) and for host vs controls and host vs substrate (Table S7).

When comparing differences between samples (*β*-diversity), we found the influence of time on sample variation to be the most prominent for host-derived samples (Aitchison distance; Fig. 2D). Although we found significant differences between sample sources (PERMANOVA *F* test *P* = 0.002, Table S8), variation over time was only identified for host and substrate samples (PERMANOVA *F* test *P* < 0.05 for Bray-Curtis, Unifrac, and Aitchison distance; Fig. S7; Table S10). Time points 66 and 186 h showed significant dissimilarities across sample sources (Table S9). For several cases, pairs of host and substrate samples from the same plate existed so that compositional distances could be compared directly between the paired samples. This allowed us to assess the dynamics of community composition between conditions. We observed that differences between host samples and controls increased over time (Fig. 2F; Fig. S8b). For host and substrate samples, Bray-Curtis and Unifrac distances indicated that they initially diverge in composition but, after 90 h, become more similar again (Fig. S8b; Fig. 2E).

We performed a differential abundance analysis using DEseq2 based on a negative binomial generalized linear model with time and source as variables to identify bacterial strains whose growth showed variation. The results were summarized in a “heat tree” that depicts differentially abundant taxa for host vs control and host vs substrate. When comparing control vs substrate samples, we did not find differentially abundant taxa (Fig. 2B). In agreement with previous studies, *Ochrobactrum* (MYb71/MYb49) and *Enterobacter* (CEent1) strains were specifically enriched in samples obtained from within the host in all comparisons. *Chyseobacterium* (MYb328) and *Pseudomonas* (MYb371/MYb331/MYb330) showed higher abundances in host compared to control samples, whereas *Comamonas* (MYb396/MYb69/MYb21) and *Chryseobacterium* (MYb328) were more abundant in host compared to substrate samples. *Acinetobacter* (MYb10, MYb158, MYb191/MYb177) belonged to the most widely abundant taxon across all samples and was, together with *Sphingobacterium* (MYb388), significantly enriched in control and substrate samples. In addition, *Pseudomonas* (MSPm1), *Chryseobacterium* (JUb44), and *Sphingomonas* (JUb134) showed higher abundances in controls in contrast to host samples (Fig. 2B; Fig. S4).

In summary, we found differences in diversity between samples obtained from hosts, substrate, and controls. The effect of time was most pronounced for microbial samples obtained from the host, and host samples diverged over time compared to controls. This suggests that specific microbial taxa were enriched in the host-associated community compared to substrate and control samples.

### Stochasticity

**Fig 3:**
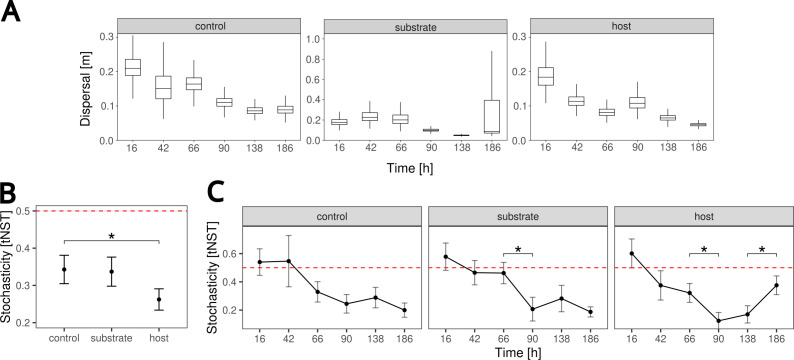
Influence of stochastic effects on microbial community dynamics. (**A**) Best-fit values of the dispersal parameter *m* of the neutral model across time and the different sample types. (**B** and **C**) Taxonomic normalized stochasticity index across time and sample types. Red dashed line (tNST = 0.5) indicates the transition of the influence of stochastic (tNST > 0.5) to deterministic (tNST < 0.5) processes in community assembly. Significant differences in tNST were observed between sample types (B) and across specific adjacent time points (C).

We next assessed to what extent deterministic and stochastic processes have shaped the assembly of microbial communities across time points and the three sample types (host, the host-associated substrate, and the host-free controls). We first took Hubbell’s neutral model to investigate the effect of stochastic effects. In a previous study, we observed low neutrality for the microbial community assembly of *C. elegans* compared to other organisms (goodness-of-fit *R*^2^ = 0.44). Since the fit of the neutral model is based on the long-term equilibrium state, we hypothesized that the microbial community in the short-lived nematode might still be in a transient state on its way to a more neutral composition, thereby underestimating neutral effects (17). Therefore, in the current study, we characterized the microbial community dynamics over the considered time points of *C. elegans* lifetime and tested whether neutrality increases across time. Surprisingly, we found that the neutral expectation fitted the observed community abundance data very well (0.8 < *R*^2^ < 0.95) regardless of the sample source, indicating that the data can be explained by a neutral model (Fig. S10). Although we did not find a continuous increase in neutrality, the dispersal parameter *m* of the neutral model decreased over time and differed between sample sources (Fig. 3A). Lower values for the dispersal parameter at later times could indicate less mixing between samples, leading to more variance between communities. However, the neutral model analysis comes with the limitation that it is based on the presence and absence of taxa, while most samples of our study continuously contained nearly all of the CeMbio43 taxa and only their relative abundance varied. Because of this limitation, we further assessed the role of stochastic effects with the taxonomic normalized stochasticity index (tNST). The tNST quantifies the effect of deterministic (tNST<0.5) and stochastic effects (tNST >0.5) based on β-diversity. The tNST changed over time for all conditions, starting with higher values (tNST >0.5) followed by a decrease, most prominently for host samples (tNST <0.2 after 90 h), and a late increase observed only for host samples (tNST ≈ 0.4 after 186 h; Fig. 3C). In general, host samples showed significantly lower stochasticity values when compared to controls (bootstrapping test *P* < 0.05; Fig. 3B). Taken together, contrary to our original hypothesis, stochasticity rather decreases across time, even if there is a slight reversal at the late time points for the host samples. This suggests that non-stochastic processes may shape the composition of microbial communities in our experiment.

### Enrichment of host-specific functions

**Fig 4:**
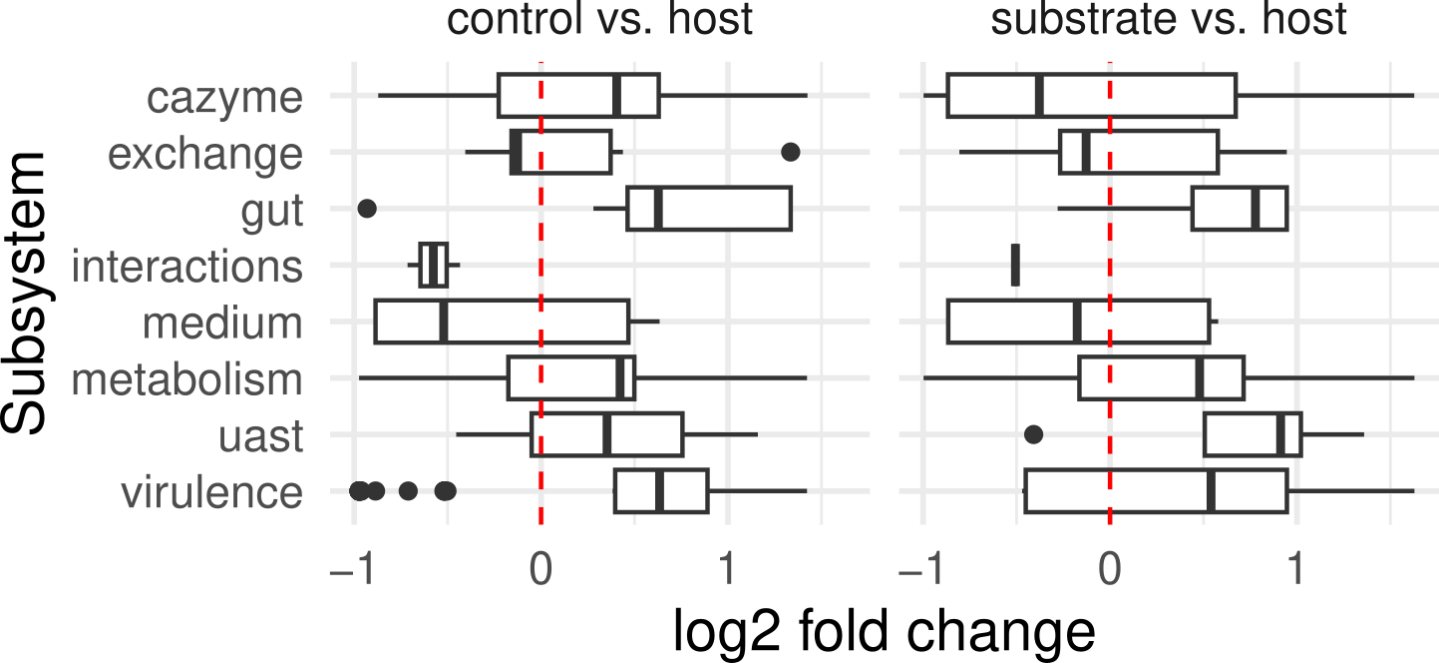
Differentially abundant functions in host compared to substrate and control microbiome. Differential abundance was assessed by DESeq2 for different functional subsystems (along vertical axis) and different comparisons of the sample types (the two panels). For example, for the comparison “substrate vs host,” positive values (log2 fold change) indicate that the host-associated microbiome showed an increased presence of the functions of the respective subsystem, while negative values a comparative decrease of the functional subsystem in the host samples. No differentially abundant functions were found for substrate compared to control microbiome. The following subsystems were considered: cazyme, carboydrate-active enzymes; exchange, carbon/energy source and metabolic byproducts; gut, gut-microbiome gene clusters; interactions, prevalence of ecological interactions based on pairwise metabolic predictions; medium, growth medium components; metabolism, MetaCyc pathways; uast, life-history strategies; virulence, virulence and resistance genes.

We identified many potential functional traits that differ between sample types. The microbial communities from *C. elegans* were significantly enriched for traits relevant to a host environment. In particular, our analyses of diversity and stochasticity already indicated deterministic effects on the microbiome assembly and differences in the dynamics between sample types (control, substrate, and host). Therefore, we wondered whether the non-stochastic effects are explained by selection of specific functions of the microbiome across time. To address this idea, we assessed the temporal changes in characteristics of the microbial community, as inferred from the microbiome abundance data in combination with the corresponding genome sequences and reconstructed metabolic models. We considered distinct types of microbiome characteristics (see Materials and Methods), including those defined by carbohydrate-active enzymes (i.e., cazyme in Fig. 4), metabolic exchange (i.e., exchange), microbiome-related gut functions (i.e., gut), pairwise interactions (i.e., interactions), growth media (i.e., medium), metabolic pathways (i.e., metabolism), adaptive strategies (i.e., universal adaptive strategy theory [uast]), and virulence factors (virulence). The predicted characteristics (presence/absence) were related to the microbial abundance data to obtain temporal information for each potential function and community.

**Fig 5:**
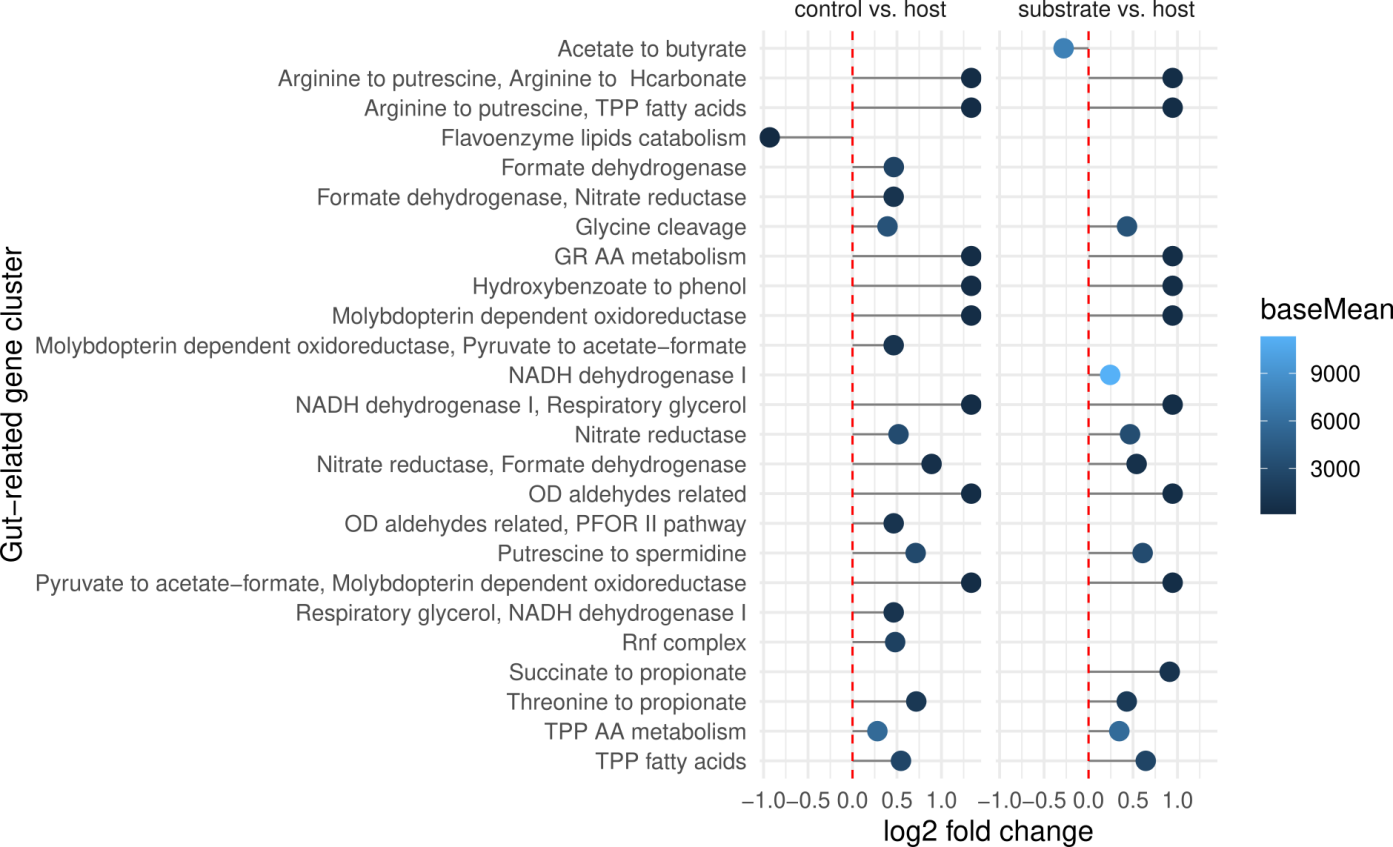
Enrichment of gut microbiome functions. Gene clusters were predicted by gutSMASH and combined with microbial abundance data. We found gut functions dominantly enriched in host samples. No differences were found for controls vs substrate. TPP, thiamine pyrophosphate; AA, amino acids; GR, glycyl radical amino acid metabolism; OD, oxidative decarboxylation; PFOR, pyruvate ferredoxin oxidoreductase; energy-converting ferredoxin, NAD+ reductase complex (Rnf).

Our analysis revealed that worm-associated microbial communities had the highest functional diversity, which increased over time (Fig. S11). Using DESeq2, we found significant trait differences for many comparisons between sample types (Fig. 4). In particular, we found glycoside hydrolases and glycosyl transferases to be differentially abundant between sample sources (Fig. S12). In addition, we inferred substrate uptake and metabolic products from metabolic models and discovered enriched capacity for tryptophan uptake and folate production in the host-associated microbiota (Fig. S13). Interestingly, gene clusters responsible for gut microbiota functions were almost exclusively increased in host samples. This result was driven by a raised abundance of genes encoding for short-chain fatty acid production, polyamine metabolism, and anaerobic energy metabolism (Fig. 5). We used the metabolic models to infer pairwise interactions between bacteria and found neutral interactions enriched in non-host samples (Fig. S14). In addition, we predicted the suitability of different growth media or nutrients from metabolic models. Compared to substrate and control samples, we found that host-enriched microbes could more often use sulfoquinovose and, to a lesser extent, N-acetylglucosamine and N-acetyl-neuraminate (Fig. S15). We further studied the presence of metabolic pathways as defined by MetaCyc (Fig. S16). Secondary metabolites degradation and carboxylate degradation were over-represented for host compared to substrate samples, whereas samples from substrates and controls showed higher levels of aromatic compound degradation (Table S11). We next assessed differential abundance of specific pathways known to be relevant for *C. elegans*. Here, vitamin B12 biosynthesis and salvage pathways were significantly more abundant in the host than in substrate samples (Fig. S20). Moreover, branched chain amino acid (BCAA) degradation pathways were enriched in the host compared to control samples (Fig. S21). In a previous study (13), we proposed to classify bacteria into competitors, stress tolerator, and ruderals, according to the universal adaptive strategy theory (47). We found an enrichment of competitive and mixed strategies in microbial samples from hosts and more stress-tolerance strategies in control and substrate samples (Fig. S17). We also explored the differential abundance of virulence genes. We identified adherence as more prevalent in substrate samples, and immune regulation, antimicrobial activity, and invasion increased for host samples (Fig. S18). Last, we checked two pathways relevant to host colonization and worm fitness as identified in our previous study (13). Consistent with our previous results, we found pyruvate fermentation to acetoin and hydroxyproline degradation almost exclusively to be differentially abundant in the host-associated microbiota (Fig. S19).

**Fig 6:**
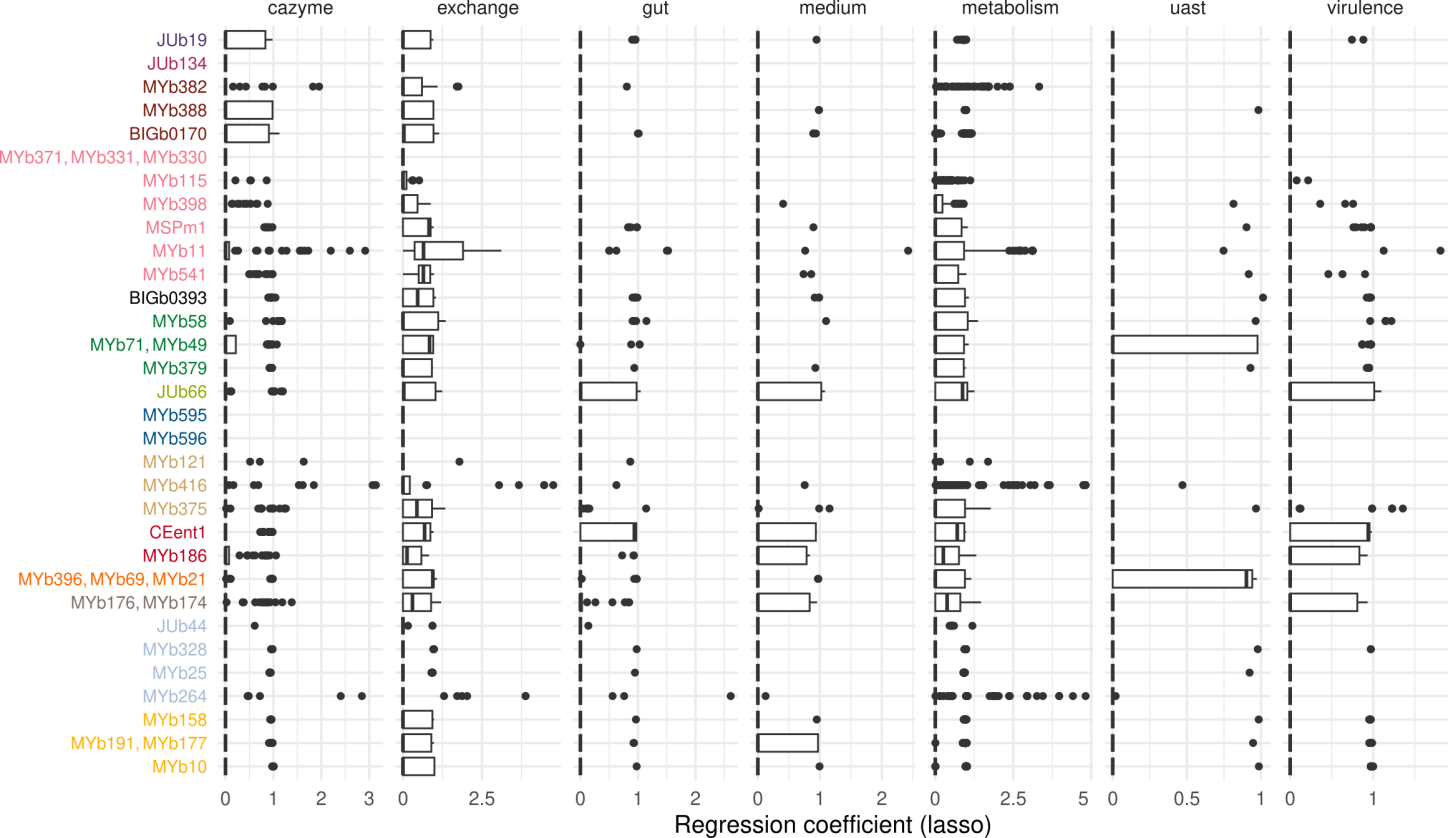
Contribution of microbial taxa to functions. The attribution of individual taxa to predicted functions was determined by regression analysis. We used a penalized regression model to estimate how well the taxon abundances can predict functional abundances. For each taxon, all coefficients from regression models summarized by subsystem are shown in boxplots. The subsystem “interactions” is not shown because of low variance. Strain codes are as in Table 1; colors indicate different genera as in Fig. 1.

For a more complete picture, we tracked the contribution of microbial species to the inferred functions. We summed up the species abundances and performed a regression analysis to account for the impact of low-abundant species on functional abundances. Although some taxa showed a more substantial contribution to some functions (as, for example, seen for the *Enterobacteriaceae* CEent1 and JUb66), we found that the enrichment of gut functions was supported by a larger variety of microbiota strains (Fig. 6; Fig. S22).

## DISCUSSION

In our study, we analyzed the assembly of a newly defined and fully sequenced microbial community across a substantial part of the lifetime of *C. elegans*. Our study is one of very few studies to characterize microbial community dynamics in this nematode across time (12). We compared microbiome characteristics across six time points and a total of 186 h for three different sample types: host, the direct substrate environment of the hosts (substrate), and an environment without nematode hosts (controls). We found that time affected *α*-diversity only for substrate and host samples. The microbial composition of host vs control samples differed with time, indicating the relevance of host factors. Strains from the genera *Ochrobactrum* and *Enterobacter* were specifically enriched in host samples. We also found less influence of stochastic effects on microbial assembly across our sample types, most prominently in host samples, possibly suggesting deterministic processes shaping microbiome composition. Finally, we inferred changes in microbial functions from the fully sequenced genomes and community composition and identified several functions, which were previously described for host-microbe interactions, to be specifically enriched in the host-associated microbial communities. By combining microbial abundance data with ecological and metabolic predictions, we addressed the compositional and physiological dynamics of microbiome assembly across a substantial part of the lifetime of the host *C. elegans*.

### Specific beneficial microbes increase in abundance in the host

In our previous study on the 12-member CeMbio community, we found *Ochrobactrum* (MYb71) and *Stenotrophomonas* (JUb19) more abundant in worms, while *Acinetobacter* (MYb10) and *Sphingobacterium* (BIGb0170) were enriched in lawns (19). The present study confirms a higher abundance of *Ochrobactrum* (MYb49/MYb71) in worms, and of *Acinetobacter* (MYb10, MYb191, MYb158) and *Sphingobacterium* (MYb388) in the host-free controls. In contrast to the earlier results, we now also found *Enterobacter* (CEent1) to be more abundant in worms but not *Stenotrophomonas*. The latter differences may be explained by the more diverse CeMbio43 community, the different *C. elegans* strain used, and/or the higher temperature of the present experiment (which was necessary to ensure worm sterility and thus unconstrained sampling of worms across its lifetime). Especially if other bacterial species of the same genus, as in the case of *Sphingobacterium*, followed the same enrichment pattern, the extended CeMbio community’s additional redundancy might help to identify the species or strains performing the same role or function. For the host-enriched *Ochrobactrum*, beneficial effects for *C. elegans*, such as ROS detoxification and increased growth, were reported before (20, 48). Host transcriptomics revealed that *Ochrobactrum* influences the nematode’s dietary response, development, fertility, immunity, and energy metabolism via GATA transcription factor (ELT-2) and C-type lectin-like genes and additional genes known to be involved in energy metabolism or fertility (49). In addition, *Enterobacter* bacteria showed consistently high prevalence in worms and provide protection against the pathogen *Enterococcus faecalis* (50, 51). The combination of known positive effects and the increased abundance of *Ochrobactrum* and *Enterobacter* in the nematode gut may suggest that *C. elegans* is able to favor presence of beneficial microbes. A dissection of the possible underlying molecular mechanisms warrants further research.

The nematode immune system possibly shapes the composition of the host-associated microbial communities. Indeed, a previous comparison of microbiome composition across a diverse set of natural *C. elegans* strains identified distinct microbiome types, most likely as a consequence of host filtering, and demonstrated a significant role of worm genetics (20). Interestingly, host filtering favored the presence of *Ochrobactrum* which was linked to the worm’s insulin-like signaling pathway (20). Although we did not find a specific *Ochrobactrum*-dominated microbiome type, *Ochrobactrum* was significantly more abundant in worms compared to controls or substrate samples (Fig. S4). Moreover, our analysis of microbiome-associated functions yielded a possible link between the presence of *Ochrobactrum* and host insulin signaling. Host samples with high *Ochrobactrum* abundance were significantly enriched for the microbiome-associated branched chain amino acid degradation pathways (Fig. S21), which were previously reported to contribute to insulin resistance (52), whereby a reduced catabolism of BCAA by the host was shown to increase the lifespan of *C. elegans* (53). If *C. elegans* may have a core microbiome is under debate (12, 54). In general, the precise definition and quantification of a core microbiome remain challenging (55). Therefore, Turnbaugh and colleagues argued that a core microbiome exists primarily on the level of shared metabolic functions (44). In our study, we introduced a microbiome community that is representative of the microbial diversity naturally encountered in *C. elegans* and that is close to what may be considered a core microbiome of this nematode. We still observed variation in microbiome composition across time and sample type. Importantly, we found the presence of host-relevant functions to be enriched in host samples and distributed across numerous microbial taxa (Fig. 6). This finding is consistent with the proposed importance of functions rather than specific taxa in defining the host-associated core microbiome (56).

### *C. elegans* microbiome assembly dynamics are shaped by selective rather than stochastic processes

Stochastic processes have been reported to shape the assembly of microbial communities in diverse contexts and host systems (57). Previously, we used the neutral model to assess stochasticity in the microbiomes of several different hosts and found the lowest neutrality for the microbiome of *C. elegans* (17). Considering the short lifespan of the nematode, we previously hypothesized that the worm-associated community might be in a transient state that can appear non-neutral temporally. In the current study, we now observed a much higher level of neutrality, even though we considered a longer time-span, with very little change in neutrality over time. However, the *α*-diversity of samples showed differences only for evenness rather than richness (Fig. 2C; Fig. S6), indicating little variation in the general presence of taxa. This may have even been expected since we used a pre-selected community of microbes known to coexist with *C. elegans* in nature. Consequently, the classical neutral approach was limited because it is based on differences in presence-absence as a function of mean relative abundances, while our data set shows little variation in presence-absence of the bacteria but instead is rather shaped by changes in relative abundances.

As an alternative to the neutral model (58), we therefore employed a null model approach, which is less influenced by presence/absence of microbes and has shown higher accuracy and precision than the neutral model in certain cases (43). The null model analysis revealed a decrease in stochasticity for all environments initially, followed by increased stochasticity for later time points only for host samples. Initially, the observed stochasticity dynamics could be a consequence of the experimental setup for which abundances were arranged artificially and did not reflect ecological conditions. Therefore, an initial decrease in stochastic effects seems reasonable to establish the more natural strain abundances and may be further influenced by selective processes in the host. The later increase of stochasticity only for host samples is generally consistent with previous reports from other animal hosts (17) and most likely reflects the aging of *C. elegans*, in which the constant pumping of new material and grinder efficiency declines after around 100 h (59). Consequently, the host is likely to lose its ability to select the presence of microbes so that microbiome composition is more strongly influenced by stochastic processes. Such an age effect has also been reported for the human microbiome, where the microbial community composition becomes less diverse and more unique with aging (60, 61). Although stochasticity was highest for host samples at later time points, the overall stochasticity (i.e., summarized across time) of host-derived samples was significantly lower than that of control and substrate samples. Given the similar starting point of stochasticity for all environmental sources, we conclude that deterministic (i.e., selective) processes may play a particular role in shaping host-associated microbial communities, consistent with the previous demonstration of host genetic influence on microbiome composition in *C. elegans* (20).

### Host-relevant traits become enriched in the *C. elegans* microbiome

We used our time-series abundance data in combination with the genome sequence-inferred metabolic characteristics to determine which microbial traits predominate in the different sample types. For the host samples, we found significantly higher abundance of many gene clusters and pathways that are known to be produced by anaerobic gut microbes and affect animal hosts (62). The presence of functions usually associated with anaerobic gut microbes further indicates the potential role of anaerobic niches in the gut of *C. elegans* (63). One example is the enriched production of short-chain fatty acids, which can have many beneficial effects in animals, including protection of *C. elegans* against neurodegeneration (64, 65). High levels of the microbial-derived short-chain fatty acid propionate can also have a toxic effect in *C. elegans,* and its degradation is linked to vitamin B12 whereby vitamin deficiency reroutes the fatty acid metabolism (66, 67). Our former study identified several members of the native microbiome of *C. elegans* to possess pathways for vitamin B12 biosynthesis (13). In agreement with these findings, we found the capacity for propionate production (by *Enterobacteriaceae*, *Comamonas*) and vitamin B12 production (by *Ochrobactrum*, *Comamonas*, *Pseudomonas*) in microbial samples from *C. elegans* enriched over time, indicating a vitamin-rich environment with B12-dependent host breakdown of propionate (67).

Another example is the higher production of polyamine spermidine and folate in the host-associated microbiome. Spermidine is known to extend the lifespan of *C. elegans,* and folate is an essential vitamin for this nematode, which affects germ cell proliferation and aging (68–70) and thus fitness-associated characteristics. In addition, the host-derived microbial samples showed higher potential to degrade N-acetylglucosamine and N-acetyl-neuraminate used for the dense glycosidation of host mucins and thus provide a source for host selection of commensal bacteria (71). However, *C. elegans’* glycans seem to lack N-acetylneuraminate (72). Nonetheless, *C. elegans* contains gangliosides (73) that could provide N-acetylglucosamine because gangliosides are glycosphingolipids that contain N-acetylglucosamine as terminal residue (74). Furthermore, we looked into microbial life histories. We encountered increased competitive strategies and competitive virulence traits in host samples (Fig. S17 and S18), which are in-line with studies emphasizing the dominance of competitive interactions in microbiomes in general (75). Finally, we confirmed pyruvate fermentation to acetoin and hydroxy-proline degradation for the host-associated microbiome, which we previously found to be associated with bacterial load and worm fitness and which could potentially be involved in worm attraction and skin microbiome interactions (13). Taken together, we identified several functions potentially critical for *C. elegans* physiology to be enriched in the worm. These findings may suggest that this nematode specifically selects bacteria providing these functions. It is an exciting area for future research to assess how exactly *C. elegans* is capable of selecting specific microbial taxa.

### Conclusion

This work introduced an approach for studying changes in microbiota-mediated functions by combining microbial abundance data with genome-inferred traits. We employed metabolic modeling, pathway analysis, and ecological concepts to infer functions potentially relevant to the microbiome of *C. elegans*. Our time-series data of a defined microbial community revealed that the host microbiome is shaped by selective rather than stochastic processes. These selective processes appear to favor an enrichment of microbiome-mediated functions in *C. elegans* that are typical for gut microbiomes and that are likely beneficial for the nematode.

## Data Availability

The raw data from genome sequencing and 16S amplicon sequencing are available at PRJNA743218. The source to reproduce figures and results can be found at Github (https://github.com/jotech/cembio-dynamics).

## SUPPLEMENTAL MATERIAL

Supplemental material mbio.00012-24-s0001.pdf Extended material and methods, Figures S1-S22, and Tables S1-S12.

ASM does not own the copyrights to Supplemental Material that may be linked to, or accessed through, an article. The authors have granted ASM a non-exclusive, world-wide license to publish the Supplemental Material files. Please contact the corresponding author directly for reuse.
