# Supplementary material for: Gut-associated functions are favored during microbiome assembly across a major part of C. elegans life
Source: mBio. 2024 Apr 18;15(5):e00012-24. doi: 10.1128/mbio.00012-24 (PMC11077962; doi:10.1128/mbio.00012-24)
Supplement: Supplemental material — Extended material and methods, Figures S1-S22, and Tables S1-S12. [file mbio.00012-24-s0001.pdf]

Supplementary file: Gut-associated functions are favored during microbiome assembly across a major part of C. elegans life

Contents

1 Extended material and methods 2
1.1 Material 2
1.1.1 Nematode strain and maintenance 2
1.1.2 Bacteria and maintenance 2
1.2 Whole genome sequence analysis of bacteria 2
1.2.1 DNA isolation and DNA sequencing 2
1.2.2 Processing of sequencing data and genome assembly 3
1.2.3 Annotation of genomic functions 3
1.3 Analysis of microbiome changes 3
1.3.1 Experiments and processing of samples 3
1.3.2 DNA isolation and 16S rDNA amplicon sequencing 4
1.3.3 Analysis of microbial census data 4
1.3.4 Stochasticity 5
1.3.5 Functional abundances 5

2 Isolation and Inoculation details 6

3 Genome assembly 7

4 Quality 16S amplicon sequencing 9
4.1 Mock community 9

5 16S census data analysis 10
5.1 Strains below detection limit 10
5.2 Filtering 10

6 Abundances 12

7 α-diversity 13

8 β-diversity 15

9 Stochasticity 17

10 Functional abundances 18

11 Software 25

1 Extended material and methods

1.1 Material

1.1.1 Nematode strain and maintenance

The *C. elegans* strain DH26 (obtained from the Caenorhabditis Genetics Center; CGC, Minnesota, USA) was used, because it is spermatogenesis defective and thus sterile at 25 °C, avoiding overlapping generations in our experiment. This strain was grown and maintained according to the standard maintenance protocol using nematode growth medium (NGM) plates seeded with *Escherichia coli* strain OP50 at 20 °C [32] Worm populations were developmentally synchronized by a bleaching protocol that simultaneously removes any associated microbes [32], leading to germfree first instar larvae (L1) for the experiment.

1.1.2 Bacteria and maintenance

We defined a community of 43 natural *C. elegans* microbiome isolates, the CeMbio43 (Table 1, Table S1), which extends the previously described *Caenorhabditis elegans* microbiome resource (CeMbio) that consists of 12 bacterial strains, representative of the naturally associated microbiome of this nematode [8]. The CeMbio43 community now includes additional taxa which we recently found to be commonly associated with *C. elegans* in nature [9, 14] and which also contains several strains for each of the abundant genera, in order to take account of strain redundancy commonly found in microbiomes. Thus, the CeMbio43 includes all 12 strains of the original CeMbio resource [8], five strains characterized previously by us in a different study [39], and another 26 strains isolated from natural *C. elegans* isolates or *C. elegans*-containing compost substrate, both collected in the Kiel Botanical Garden, Germany (Table 1, Table S1). This extended community earlier consisted of 46 isolates containing three more strains of the genera *Stenotrophomonas* (2x) and *Chryseobacterium* (1x), which we subsequently ignored because of cultivating problems and/or a potential pathogenic effect on worms. The three strains could also not be found in the 16S amplicon sequencing (relative abundance ¡0.0001). We, therefore, excluded the three strains to ensure consistency with our other work, in which the 43-member microbiome was introduced [24]. For DNA isolation and our experiments, the 43 bacterial strains of the CeMbio43 community were individually grown in 4 ml lysogeny broth (LB), using 48-Well 6 ml polypropylene storage plates and an inoculation of 4 µl bacterial stock culture (due to slow growth, 10 µl of *Gluconobacter* strains MYb592, MYb595, MYb596), followed by incubation for 2 days at 25 °C on a circular vibrating shaker at 350 rpm. Individual isolates were frozen in stocks of filter-sterilized 10 % DMSO. Each stock was only used once.

1.2 Whole genome sequence analysis of bacteria

1.2.1 DNA isolation and DNA sequencing

For the 26 bacterial strains without existing genome information (Table S2), we isolated high quality genomic DNA using the NucleoSpin Tissue Kit (Macherey-Nagel) following the support protocol for bacteria, including a RNase A digestion by incubating the samples with 0.02 mg RNase A for 20 minutes, followed by the standard protocol for human or animal tissue and cultured cells. The DNA was then eluted in 50 µl pre-warmed BE buffer and directly used for sequencing, without subsequent freezing. All steps were performed using Thermo Scientific™ ART™ Wide Bore Filtered Pipette Tips and DNA LoBind tubes (Eppendorf).

Whole genomes of all 43 strains were either obtained earlier or during this study (see Supplementary Table S1). The latter were either sequenced with PacBio and/or NextSeq. In detail, NextSeq sequencing was performed on the NextSeq 500 with the NextSeq 500/550 High Output Kit v2.5 (300 Cycles). All samples were preprocessed with the transposase Tn5 as described in Picelli et al. 2014 [25]. For PacBio sequencing, library preparation was done with the SMRTBell Express template prep kit 2.0 from Pacific Biosciences according to the protocol for microbial multiplexing libraries. The libraries were pooled based on genome size and sequenced on the Pacific

Biosciences Sequel II instrument on one SMRTCell 8M with the Sequel binding kit 2.0 and Sequel sequencing kit 2.0 (sequencing primer v4). Movie time was 30 hrs. Demultiplexing was done on CLR with Lima.

1.2.2 Processing of sequencing data and genome assembly

Raw reads from genome sequencing were filtered using fastq_illumina_filter 0.1, trimmed with PRINSEQ-lite 0.20.4 [26] and re-synchronized by repair.sh (from BBMap version 38.87) [3]. The genome assembly was done with several algorithms and piplelines for comparison: i) SPAdes v3.14.1 [1], ii) MaSuRCA 3.4.2 [38], iii) Unicycler v0.4.8 (normal and bold mode) [35], iv) shovill 1.1.0 [28], v) SKESA 2.4.0 [31]. In addition to short reads, long reads were available for some isolates (MYb115, MYb177, MYb264). For these cases, additional hybrid assemblies were obtained from SPAdes, MaSuRCA, and Unicycler.
The quality of the assembled genomes was evaluated using QUAST v5.1.0rc1 [20]. For each assembly, a score was calculated based on characteristics such as number of contigs, N50, L50, largest contig, completeness, mapped reads, properly paired reads, and N's per 100 kbp. The score was increased by 1 if the value of a characteristic belonged to the best 25% of all assemblies for an organisms. The characteristics 'completeness' and 'longest contig' were considered as most important and the score was increased by 2 if their value belonged to the best 5%. All assembly contigs were scrutinized for potential contamination using blobtools 1.1.1 [15]. For this means, short raw reads were sorted with samtools 1.10 [17] and mapped against the assembled contigs by BWA [16]. The assembled contigs were aligned against the nucleotide database (NCBI nt, 09/2019) employing blastn [5]. As a consequence, a contig classified by blobtools as Nematode was removed for MYb398. Finally, basic polishing was done based on coverage length GC plots as proposed by [11]. Contigs were filtered by length ($> 500bp$), coverage (> 5), and GC (< 0.95) and the remaining contigs were considered as the final genomic sequence (Figure S1. Whenever possible, processes were parallelized with gnu parallel [34]. Details on different parameters used for programs are listed in Table S12.

1.2.3 Annotation of genomic functions

For all bacterial strains, we used the genome sequencing data to annotate genomic functions. In detail, the metabolic pathways were predicted, network models reconstructed, growth media inferred, and carbon source and fermentation products determined using gapseq 1.1 (Sequence DB: 1139b8e, bitscore cutoff 150). Virulence, resistance genes, and plasmids were scanned with abricate 1.0.1 [27] using the databases vfdb 2018-Jul-16 [7], plasmidfinder 2022-Mar-8 [6], resfinder 2022-Mar-8 [36], megares 2022-Mar-8 [10]. Gut microbiome specific gene clusters were identified by gutSMASH 1.0.0-1555cd7 [23]. For CAzyme annotation, dbCAN 3.0.2 was used [37]. The microbial interactions were simulated by a pairwise comparison of single vs. co-growth rate assuming TSB growth medium with BacArena 1.8.2 [2]. Details on different parameters used for programs are listed in Table S12.

1.3 Analysis of microbiome changes

1.3.1 Experiments and processing of samples

Temporal changes in microbiota composition were assessed for nematode populations, the directly connected NGM plate environments (labelled 'substrate'), and the control NGM plates without worms (labelled 'control') in ten replicates and across six time points. DH26 *C. elegans* populations were synchronized directly before start of the experiment. The bacterial inoculum was prepared by mixing 200 to 2000 µl of individual bacterial cultures, ensuring similar cell numbers per strain (Table S1). The inoculum was washed three times with sterile phosphate-buffered saline (PBS), followed by inoculation of NGM plates with 500 µl of the mix at OD(600 nm) 1 (t0) and subsequent incubation for two hours at 25 °C (t1). Samples from t0 and t1 were frozen for later analysis. Around 500 L1 nematodes were added per plate and maintained at 25 °C. Nematodes and bacterial lawns were sampled after 16 h (t2), 42 h (t3), 66 h (t4), 90 h (t5), 138 h (t6), and 186 h (t7).

At each time point, nematodes and bacterial lawns were washed off the plates. Worms were separated from environmental microbiota using a modification of a previously published washing protocol [22]. In detail, 2 ml of each sample was distributed over the tops of five sterile filter tips (PreCision SafeSeal LC2695, Biozym) placed on specifically designed autoclaved aluminium boxes [22]. The filter tips were sealed with aluminium foil to prevent spill-over and cross-contamination. The boxes were centrifuged at 3000 rpm for 1 min, permitting isolation of all *C. elegans* developmental stages from the filters of the tips, while the wash-through including the environmental bacteria is collected in the wells of the aluminium box. The wash-through of each individual sample was then transferred to a sterile 1.5 ml tube. The tubes were centrifuged at 5000 rpm for 10 min, the supernatant was discarded, and the pellet was resuspended in 195 µl 5xGoTaq buffer (Promega, Madison, USA) and 5 µl Proteinase K and frozen at -80 °C until further use.
The worms from each individual sample were pooled from the filters by resuspension with 250 µL filter-sterilized M9-buffer incl. 0.025% Triton X-100 (M9-T) and collected in a sterile 1.5 ml reaction tube. The worms were allowed to settle, followed by transfer of 100 µl worm pellet to a new tube including 100 µl 10 mM tetramisole hydrochloride (Sigma-Aldrich Chemie GmbH, Steinheim, Germany) in M9-T. Tetramisole paralyzes the worms and thus prevents loss of gut-associated bacteria. Any surface adherent bacteria were removed by incubating the worms with 200 µL 2% alkaline hypochloride solution in M9-T for 2 min [8]. We checked the tubes individually to avoid disruption of the worm tissue. Approximately 300 µl of the supernatant was removed and the samples were washed five times with 1 ml filter-sterilized PBS incl. 0.025 % Triton X (PBS-T). The number of worms per tube was determined and the worms were transferred to a new tube containing 17 autoclaved 1 mm zirconium beads, topped up to 400 µl with PBS-T and disrupted for 3 min at 30 Hz (Bead Ruptor 96; Omni International, Inc. Kennesaw, USA). Samples were centrifuged at 5000 rpm for 10 min, the supernatant was discarded, and the pellet was resuspended in 195 µL 5xGoTaq buffer and 5 µL Proteinase K and frozen at -80 °C until further use.

1.3.2 DNA isolation and 16S rDNA amplicon sequencing

DNA was extracted with the Nucleo Spin® 96 Tissue Kit (Macherey-Nagel, Düren, Germany) with the following modifications: After thawing the samples, 50 µl of each sample were mixed with 170 µl Lysis buffer (150 µl buffer T1, 20 µl proteinase K) and disrupted for 3 min at 30 Hz using the Bead Ruptor 96. Following incubation at 56 °C overnight, 300 µl lysate were transferred to the wells of a DW block and mixed with 300 µl buffer BQ1 and 300 µl 99 % ethanol. Mock community dilution series (ZymoBIOMICS Microbial Community Standard; Catalog No. D6300)) and negative controls were included on each plate. The 16S library was prepared using the 341F (5'-CCTACGGGNGGCWGCAG-3') and 806R primer (5'-GACTACHVGGGTATCTAATCC-3') covering the V3–V4 region of the 16S rRNA gene. Libraries were sequenced on the Miseq platform using the MiSeq Reagent Kit 2 $\times$ 300 bp (Illumina).

1.3.3 Analysis of microbial census data

We processed the obtained 16S amplicon sequencing data using the standardized amplicon sequencing pipeline nf-core/ampliseq 2.1.1 [33]. Sample sequences were inferred by DADA2 [4] using a truncation length of 280/230 for forward and reverse reads. Species were initially assigned employing the SILVA database (v138.1) for filtering, and afterwards, filtered sequences were assigned by alignment of reference 16S sequences for the CeMbio43 community inferred from the genomes (blastn -perc_identity 99 -qcov_hsp_perc 95).

The microbial composition data (ASV and taxonomic table) was analyzed with phyloseq 1.38.0 [19]. Low biomass samples are prone to index switching, a technical bias that hampers the interpretation of those samples [13]. When we compared the frequency of read counts per sample, we found a clear separation between samples with a low number of reads and the remaining samples with a high number of reads, with the low-reads samples being evenly spread across sample types (Figure S3). Therefore, to minimize any risk of artifacts, we removed

samples with less than 12,0000 reads. ASVs with uncharacterized phylum or ASVs belonging to phyla with low prevalence (< 10) were also removed, which was also the threshold below which Cyanobacteria occurred (Table S4). ASVs were agglomerated by matching them against 16S reference sequences to identify individual CeMbio community members. Some strains of the CeMbio43 community showed very high similarities in the V3V4 region of their rRNA to one another, which rendered them indistinguishable for the 16S analysis (Table S3). These highly similar strains were on purpose part of the CeMbio43 community to acknowledge high redundancy of taxonomically and functionally highly similar strains, often found in microbiomes (Figure 1). For all cases, where strains could not be distinguished, we used combined strain names. ASVs that could not be matched to members of the CeMbio43 community were labeled as 'other' and kept during the analysis, therey accounting for a certain level of random noise. The α diversity was determined by the function `plot_richness` of phyloseq. DESeq2 1.34.0 was used to find differentially abundant species [18] that were visualized by heat trees using the R package metacoder 0.3.5.001 [12].

1.3.4 Stochasticity

To assess the neutrality of the samples the expected long-term distribution of a neutral model was fitted to the sample abundance data [29, 30]. The goodness-of-fit measure R^2 then quantifies how well the abundance data conforms to a distribution expected from purely neutral dynamics. The Python script that was used is available at `www.github.com/misieber/neufit`. To evaluate the impact of stochastic processes on community assembly, a null model called Normalized Stochasticity Ratio (NST) was evaluated using the R package NST 3.1.10 [21] (function `tNST`). Sample source was used as treatment parameter (`treat.t`) and the null model with dirichlet distribution was used as it is the default for relative or proportional abundance data. p-values for comparisons were determined by bootstrapping using the function `nst.boot` with 1000 randomizations (output variable `p.count`).

1.3.5 Functional abundances

We combined the microbial 16S data with the functions predicted from genomic analysis and modeling (1.2.3). For each organism, the presence (true/false) of a particular function indicated if the organism's abundance contributed to the abundance of the function. Finally, by adding up the abundances of all species in a sample for which the presence of a function was predicted, the overall abundance of the function was determined. Like the species-level analysis, DESeq2 1.34.0 was then used to identify differentially abundant functions.

2 Isolation and Inoculation details

Table S1: Isolation details for species included in the CeMbio43 bacterial community.

Species	ID	Lab origin[a]	Source[b]	Worm strain[c]	Volume[d]
Acinetobacter guillouiae	MYb10	Schulenburg Lab, Kiel, Germany	worm	MY316	1000 µl
Pseudomonas lurida	MYb11	Schulenburg Lab, Kiel, Germany	worm	MY316	2000 µl
Comamonas sp. B-9	MYb21	Schulenburg Lab, Kiel, Germany	worm	MY316	2000 µl
Chryseobacterium culicis	MYb25	Schulenburg Lab, Kiel, Germany	worm	MY316	500 µl
Ochrobactrum anthropi	MYb49	Schulenburg Lab, Kiel, Germany	worm	MY316	500 µl
Ochrobactrum pituitosum	MYb58	Schulenburg Lab, Kiel, Germany	worm	MY316	200 µl
Comamonas sp. TK41	MYb69	Schulenburg Lab, Kiel, Germany	worm	MY316	500 µl
Ochrobactrum vermis	MYb71	Schulenburg Lab, Kiel, Germany	worm	MY316	500 µl
Pseudomonas fluorescens	MYb115	Schulenburg Lab, Kiel, Germany	worm	MY379	2000 µl
Erwinia billingiae	MYb121	Schulenburg Lab, Kiel, Germany	worm	MY379	1000 µl
Acinetobacter johnsonii	MYb158	Schulenburg Lab, Kiel, Germany	compost	NA	2000 µl
Enterobacter ludwigii	MYb174	Schulenburg Lab, Kiel, Germany	compost	NA	500 µl
Enterobacter ludwigii	MYb176	Schulenburg Lab, Kiel, Germany	compost	NA	200 µl
Acinetobacter sp. B2070	MYb177	Schulenburg Lab, Kiel, Germany	compost	NA	2000 µl
Sphingobacterium faecium	MYb181	Schulenburg Lab, Kiel, Germany	compost	NA	500 µl
Enterobacter sp. 638	MYb186	Schulenburg Lab, Kiel, Germany	compost	NA	500 µl
Acinetobacter sp. 4D-W-22	MYb191	Schulenburg Lab, Kiel, Germany	compost	NA	2000 µl
Enterobacter cloacae	CEent1	Shapira Lab, Berkeley, CA, US	worm	N2	200 µl
Pseudomonas berkeleyensis	MSPm1	Shapira Lab, Berkeley, CA, US	worm	N2	2000 µl
Chryseobacterium sp.	MYb264	Schulenburg Lab, Kiel, Germany	worm	MY2771	500 µl
Chryseobacterium sp.	MYb328	Schulenburg Lab, Kiel, Germany	worm	MY2768	200 µl
Pseudomonas sp.	MYb330	Schulenburg Lab, Kiel, Germany	worm	MY2768	200 µl
Pseudomonas sp.	MYb331	Schulenburg Lab, Kiel, Germany	worm	MY2768	2000 µl
Pseudomonas sp.	MYb371	Schulenburg Lab, Kiel, Germany	worm	MY2768	2000 µl
Erwinia sp.	MYb375	Schulenburg Lab, Kiel, Germany	worm	MY2769	500 µl
Ochrobactrum sp.	MYb379	Schulenburg Lab, Kiel, Germany	worm	MY2769	2000 µl
Sphingobacterium sp.	MYb382	Schulenburg Lab, Kiel, Germany	worm	MY2769	200 µl
Sphingobacterium sp.	MYb388	Schulenburg Lab, Kiel, Germany	worm	MY2769	1000 µl
Comamonas sp.	MYb396	Schulenburg Lab, Kiel, Germany	worm	MY2769	2000 µl
Pseudomonas sp.	MYb398	Schulenburg Lab, Kiel, Germany	worm	MY2769	200 µl
Erwinia sp.	MYb416	Schulenburg Lab, Kiel, Germany	worm	MY2771	1000 µl
Erwinia sp.	MYb535	Schulenburg Lab, Kiel, Germany	worm	MY2768	2000 µl
Pseudomonas sp.	MYb541	Schulenburg Lab, Kiel, Germany	worm	MY2768	2000 µl
Lelliottia amnigena	JUb66	Félix Lab, Paris, France	rotting apple	NA	2000 µl
Pantoea nemavictus	BIGb0393	Samuel Lab, Houston, TX, US	rotting petasites stem	NA	2000 µl
Sphingomonas molluscorum	JUb134	Félix Lab, Paris, France	worm	NA	2000 µl
Stenotrophomonas indicatrix	JUb19	Félix Lab, Paris, France	rotting pear	NA	2000 µl
Comamonas piscis	BIGb0172	Samuel Lab, Houston, TX, US	worm	NA	200 µl
Sphingobacterium multivorum	BIGb0170	Samuel Lab, Houston, TX, US	worm	NA	200 µl
Chryseobacterium scophthalmum	JUb44	Félix Lab, Paris, France	rotting apple	NA	500 µl
Gluconobacter wancherniae	MYb592	Schulenburg Lab, Kiel, Germany	rotting apple	NA	2000 µl
Gluconobacter cerinus	MYb595	Schulenburg Lab, Kiel, Germany	rotting apple	NA	2000 µl
Gluconobacter albidus	MYb596	Schulenburg Lab, Kiel, Germany	rotting apple	NA	2000 µl

[a]lab which originally isolated the bacterium

[b]isolation of the bacterium from either natural *C. elegans* or *C. elegans*-containing substrate

[c]Information on the specific *C. elegans* strain, from which the bacteria were isolated; NA, not applicable.

[d]Volume of an overnight culture, which was used to prepare the CeMbio43 mixture, which was used to inoculate the plates for the experiment.

3 Genome assembly

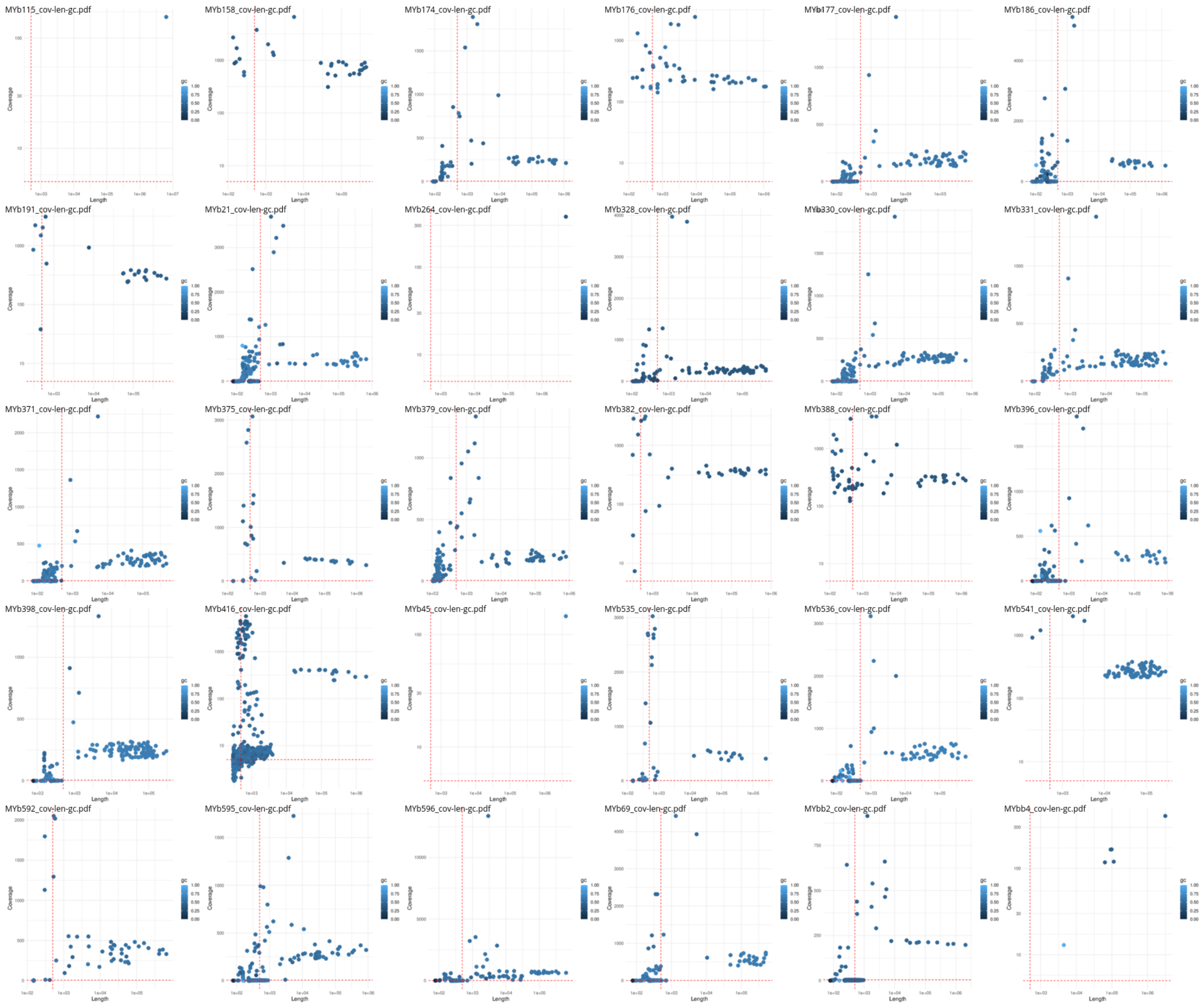

Figure S1: Coverage-length-GC plots of the assembled genomic contigs were used for basic polishing. We excluded contigs that had length < 500, coverage < 5, or GC > 0.95 (red lines indicates these thresholds used for filtering).

Table S2: Overview of newly assembled genomes. In total, 26 genomes were obtained using different assemblers. The program with the best assembly is shown together with the quality properties of the assembled genome.

ID	Organism	Assembler	N50	L50	Contigs	Length	Completeness	Genes
MYb115	*Pseudomonas fluorescence*	unicycler	6342245	1	1	6342245	100.00	5767
MYb158	*Acinetobacter johnsonii*	unicycler	346291	5	23	3599479	97.30	3425
MYb174	*Enterobacter ludwigii*	shovill	482322	4	29	4642087	97.97	4325
MYb176	*Enterobacter ludwigii*	unicycler	560529	3	37	4632577	97.97	4329
MYb177	*Acinetobacter sp. B2070*	spades	3551026	1	2	3566943	100.00	3311
MYb186	*Enterobacter sp. 638*	spades	408218	4	26	4824469	98.65	4505
MYb191	*Acinetobacter sp. 4D-W-22*	masurca	338615	4	21	3528846	96.62	3372
MYb21	*Comamonas sp. B-9*	spades	312419	7	36	5363665	97.30	4802
MYb264	*Chryseobacterium sp.*	unicycler	5234367	1	1	5234367	93.24	4562
MYb328	*Chryseobacterium sp.*	shovill	163502	11	60	5570205	93.92	5006
MYb330	*Pseudomonas sp.*	spades	206174	11	65	6101970	99.32	5563
MYb331	*Pseudomonas sp.*	shovill	168801	12	71	6097985	99.32	5557
MYb371	*Pseudomonas sp.*	spades	223371	10	53	6101404	99.32	5551
MYb375	*Erwinia sp.*	masurca	706071	2	24	4907227	96.62	4512
MYb379	*Ochrobactrum sp.*	shovill	524112	4	47	5072635	96.62	4855
MYb382	*Sphingobacterium sp.*	masurca	530684	4	29	4738001	93.24	4023
MYb388	*Sphingobacterium sp.*	unicycler	794901	3	29	6215939	93.24	5300
MYb396	*Comamonas sp.*	spades	488653	4	23	5338904	97.30	4744
MYb398	*Pseudomonas sp.*	spades	92388	21	105	5492602	100.00	5032
MYb416	*Erwinia sp.*	masurca	601712	3	443	5789730	94.59	5710
MYb535	*Erwinia sp.*	masurca	2570355	1	25	4921382	93.92	4604
MYb541	*Pseudomonas sp.*	unicycler	109376	20	82	6347450	100.00	5874
MYb592	*Gluconobacter wancherniae*	masurca	409404	4	40	3234488	95.27	3055
MYb595	*Gluconobacter cerinus*	spades	371515	3	48	3591761	95.27	3345
MYb596	*Gluconobacter albidus*	spades	191545	5	51	3298982	96.62	3068
MYb69	*Comamonas sp. TK41*	spades	274857	7	28	5262490	97.30	4659

4 Quality 16S amplicon sequencing

4.1 Mock community

In Figure S2, the experimentally found composition of the Zymo mock community is compared with theoretical values provided by the manufacturer (ZymoBIOMICSTM Microbial Community Standard. Catalog No. D6300). *Salmonella* was only found at low abundances ($\leq 5\%$, category: 'x_other(low)') and *Lactobacillus* was classified as *Limosilactobacillus*. *Enterococcus* was found in two samples. One sample was removed because of a very low read count (< 100).

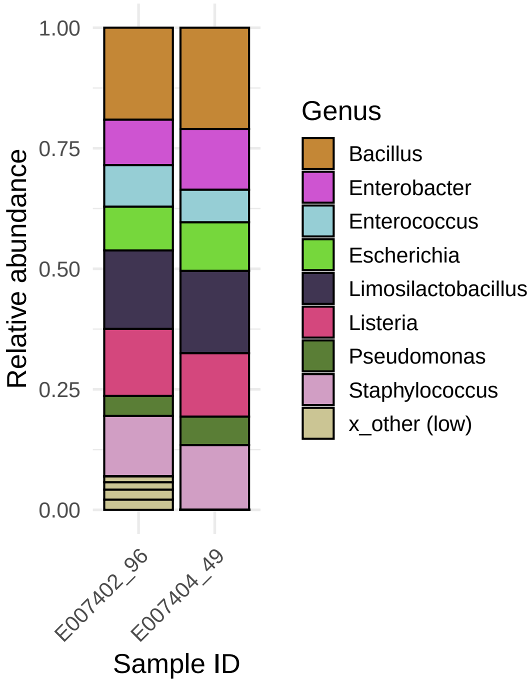

Figure S2: Zymo mock community: Experimentally found ASVs of a defined community was used for quality control. Label 'x_other (low)' indicates taxa with low maximal abundance ≤ 0.05.

5 16S census data analysis

5.1 Strains below detection limit

Table S3: CeMbio43 strains below the detection limit. The table lists taxa for which the 16S amplicon sequencing could not detect certain strains because V3V4 did not allow to distinguish several strains from each other and therefore, we only analyzed them as one unit.

Code	Merged Strains
MYb191.177	MYb191, MYb177
MYb330.331.371	MYb371, MYb331, MYb330
MYb21.69.396	MYb396, MYb69, MYb21
MYb49.71	MYb71, MYb49
MYb174.176	MYb176, MYb174

5.2 Filtering

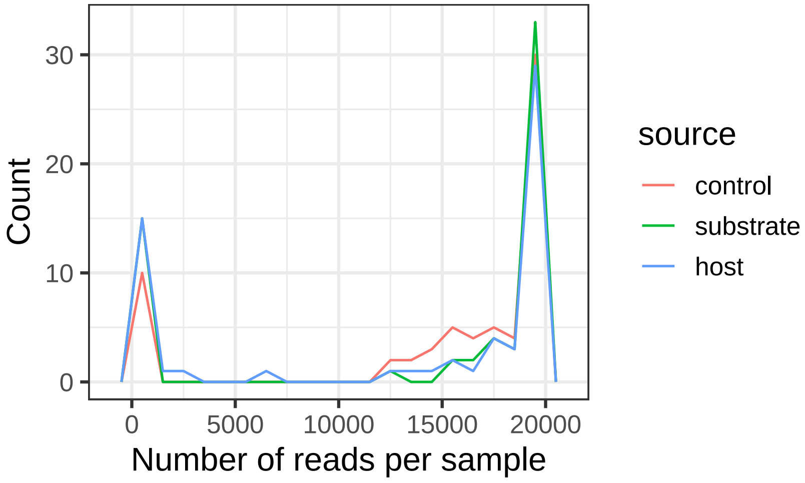

Figure S3: Frequency of samples with various read counts. All samples and their number of reads are shown. The figure displays a group of low-read-count samples and a group of high-read-count samples. Samples with 20000 or more reads were grouped.

Table S4: Prevalence was compared on the phylum level to identify a threshold for filtering. We removed ASVs belonging to phyla with very low prevalence (prevalence sum < 10).

Phylum	Prevalence (mean)	Prevalence (sum)	Abundance
Proteobacteria	12.5044	4239	0.7271
Bacteroidetes	103.6154	1347	0.2679
Bacteroidota	6.7720	1307	0.0048
Firmicutes	0.4189	31	0.0001
Actinobacteriota	0.6600	33	0.0001
Cyanobacteria	0.7000	7	0.0000
Bdellovibrionota	0.5000	1	0.0000
Chloroflexi	0.5000	1	0.0000
Patescibacteria	0.7143	5	0.0000
Myxococcota	1.0000	1	0.0000
Verrucomicrobiota	1.0000	1	0.0000
Planctomycetota	1.0000	1	0.0000
Acidobacteriota	0.7500	3	0.0000
Spirochaetota	0.0000	0	0.0000
Nitrospirota	1.0000	1	0.0000
Deinococcota	0.0000	0	0.0000
Gemmatimonadota	1.0000	1	0.0000

6 Abundances

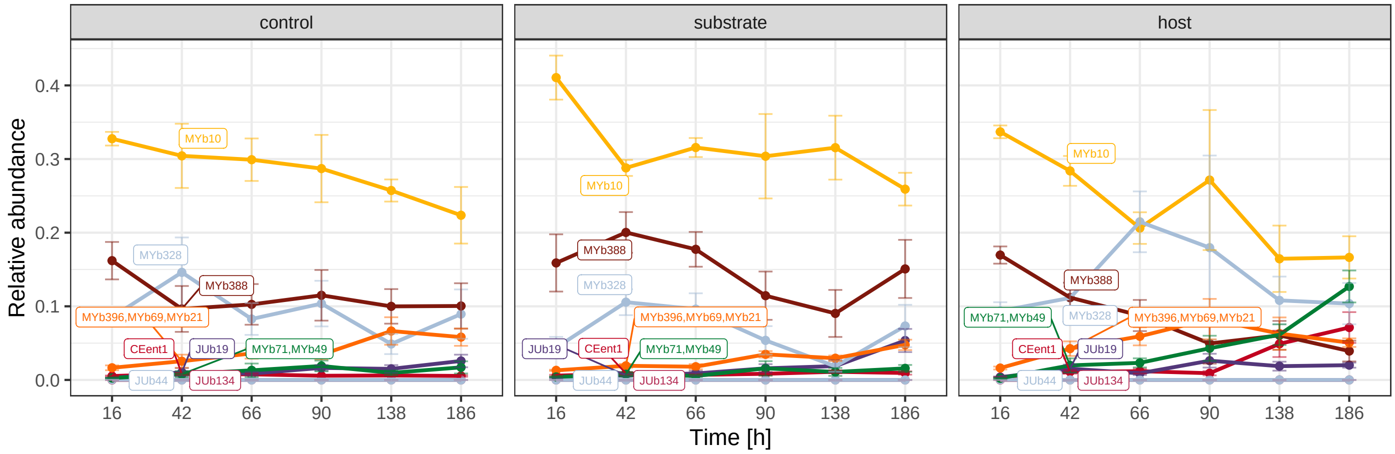

Figure S4: Relative abundances of taxa found to be differentially abundant between samples types (host, substrate, control). For each time point, mean relative abundances and standard errors are shown.

7 α-diversity

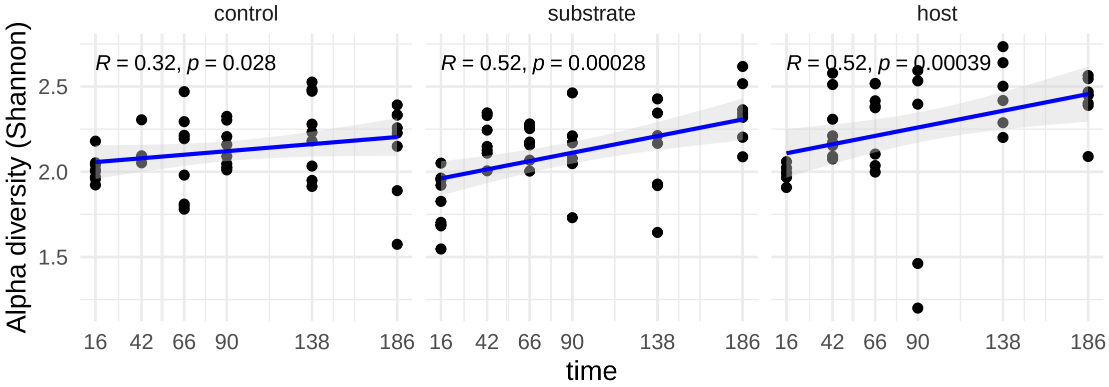

Figure S5: Shannon α-diversity for sample sources is shown, including the linear regression (blue) and Spearman correlation test to visualize the diversity trend over time.

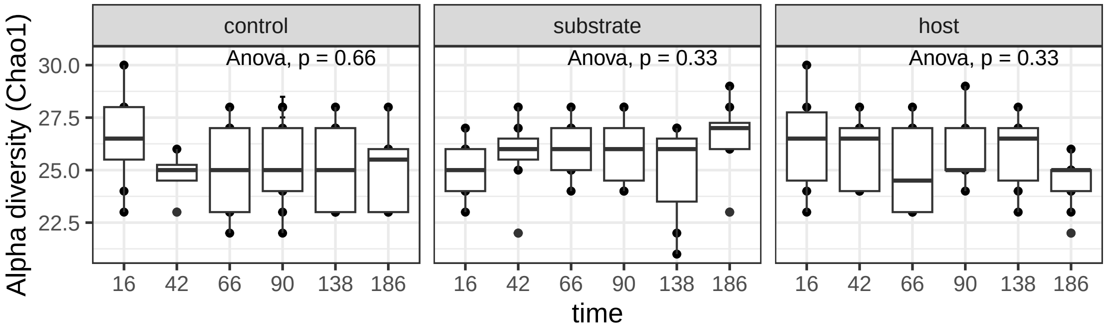

(a) Chao1

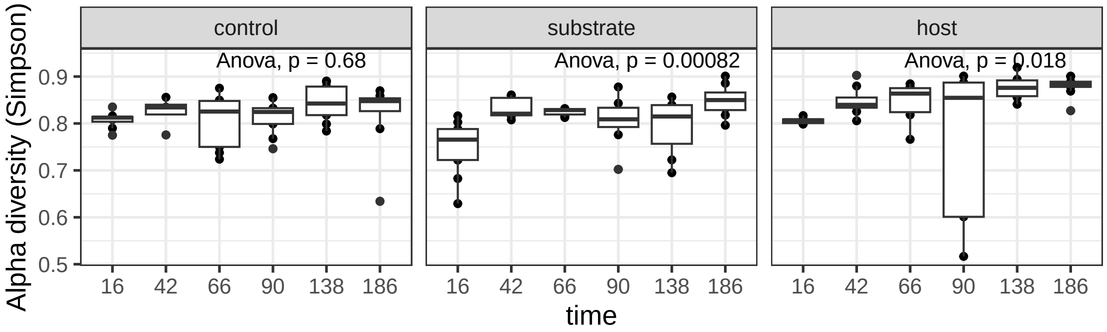

(b) Simpson

Figure S6: α-diversity of filtered and agglomarated microbial samples on taxon level. Top row shows richness (Chao1) and bottom row focuses on evenness (Simpson)

Table S5: ANOVA test of Shannon α-diversity checking for sample sources, time, and the interaction between sample source and time.

	Df	Sum Sq	Mean Sq	F value	Pr(>F)
source	2	0.6495	0.3248	6.640	0.0019
time	5	1.8774	0.3755	7.677	0.0000
source:time	10	0.7617	0.0762	1.557	0.1282
Residuals	116	5.6737	0.0489		

Table S6: Tukey Honest Significant Difference test for Shannon α diversity to check for differences between time points. Only significant pairs are shown (p adj < 0.05).

time	diff	lwr	upr	p adj
42-16	0.2766	0.0678	0.4853	0.0027
66-16	0.2484	0.0519	0.4449	0.0049
138-16	0.3186	0.1178	0.5194	0.0001
186-16	0.3737	0.1792	0.5683	0.0000
186-90	0.2036	0.0043	0.4029	0.0423

Table S7: Tukey Honest Significant Difference test for Shannon α diversity to check for differences between sample sources.

source	diff	lwr	upr	p adj
substrate-control	-0.0158	-0.1403	0.1088	0.9515
host-control	0.1417	0.0149	0.2685	0.0244
host-substrate	0.1575	0.0294	0.2856	0.0116

8 β-diversity

Table S8: PERMANOVA test of differences between samples sources (Aitchison distance, adonis2 function of R packages vegan, 10000 permutations).

	Df	SumOfSqs	R2	F	Pr(>F)
source	2	292.9	0.042	2.869	0.001
Residual	131	6686.4	0.958		
Total	133	6979.3	1.000		

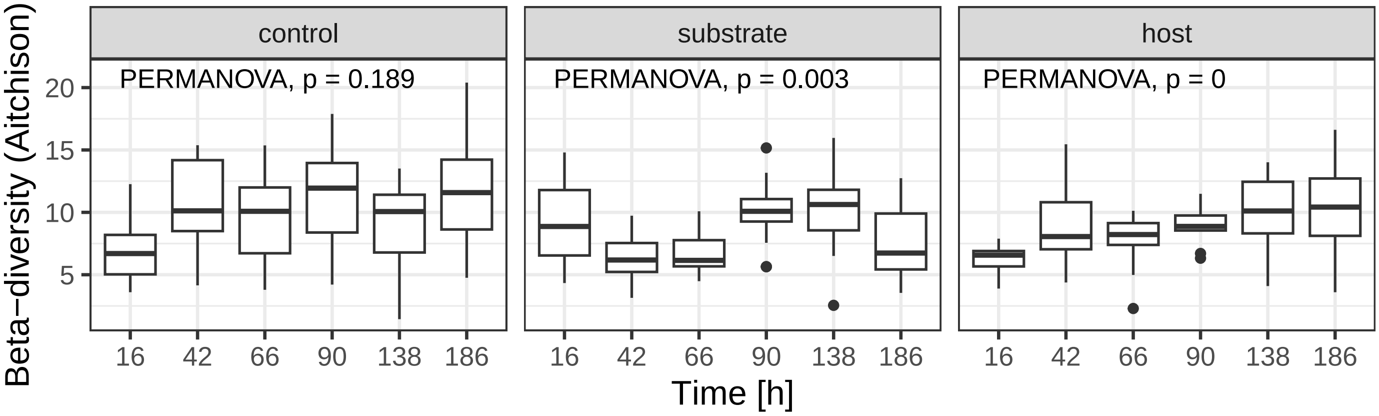

Figure S7: Time variation of the β-diversity for each sample type. Aitchison distance was used to calculate dissimilarity. The significance of time variation was determined by a PERMANOVA test (adonis2 function of R packages vegan, 10000 permutations).

Table S9: Dispersion test and PERMANOVA test of differences for each time point (Bray-Curtis, Aitchison, and weighted Unifrac distance; adonis2 and betadisper function of R package vegan, 10000 permutations). p-values for each test are shown.

time	dispersion test (bray)	permanova test (bray)	dispersion test (aitchison)	permanova test (aitchison)	dispersion test (unifrac)	permanova test (unifrac)
16	0.1056	0.1028	0.1692	0.0589	0.2003	0.4251
42	0.7969	0.1536	0.4181	0.7873	0.6204	0.1691
66	0.2410	0.0092	0.1805	0.0700	0.1623	0.0080
90	0.8060	0.7112	0.2804	0.8509	0.6456	0.9090
138	0.7258	0.0202	0.9031	0.0855	0.7036	0.0507
186	0.3683	0.0018	0.2087	0.0108	0.7671	0.0091

Table S10: Dispersion test and PERMANOVA test of differences for each sample source (Bray-Curtis, Aitchison, and weighted Unifrac distance; adonis2 and betadisper function of R package vegan, 10000 permutations). p-values for each test are shown.

source	dispersion test (bray)	permanova test (bray)	dispersion test (aitchison)	permanova test (aitchison)	dispersion test (unifrac)	permanova test (unifrac)
control	0.0193	0.3620	0.3078	0.1848	0.3060	0.6898
substrate	0.0279	0.0112	0.0975	0.0032	0.1418	0.0315
host	0.0059	0.0001	0.1412	0.0001	0.0074	0.0003

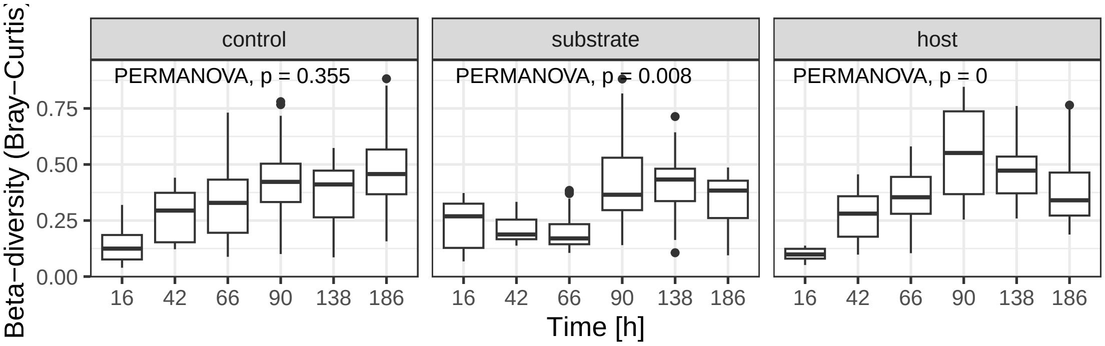

(a)

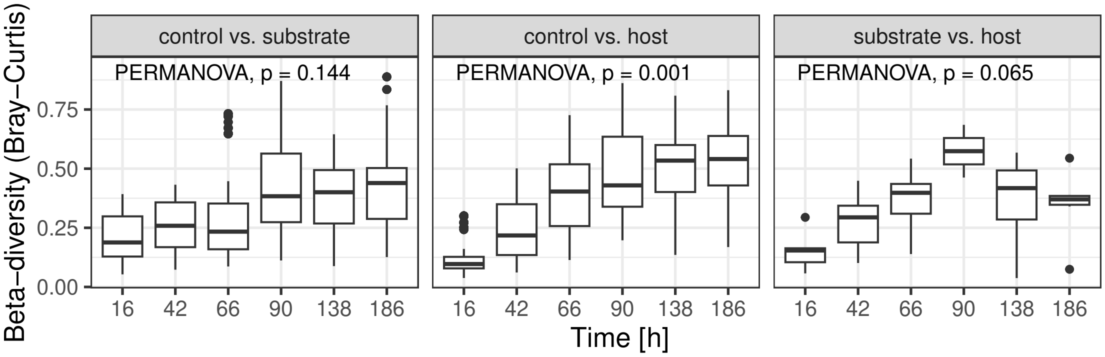

(b)

Figure S8: β-diversity using Bray-Curtis distance. Top row shows the results for each sample source over time. The bottom row shows differences compared between sample types. For comparing host-substrate samples, pairs from the same replicate were available, whereas for comparing host-control and substrate or control, pairs were randomly associated (100 repetitions), and the mean p-value is shown. The significance of time variation was determined by a PERMANOVA test (adonis2 function of R packages vegan, 10000 permutations).

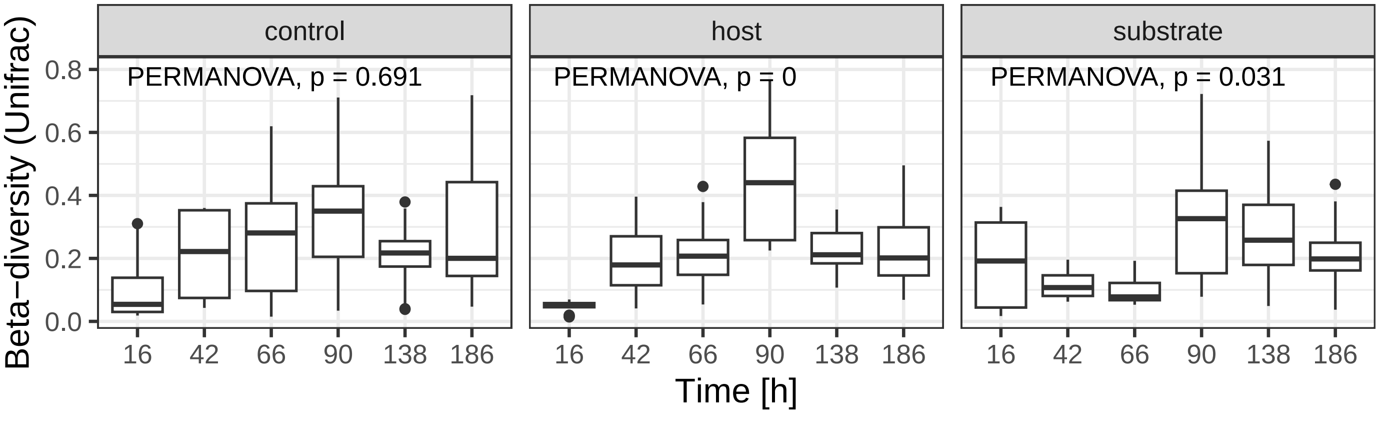

Figure S9: β-diversity using weighted Unifrac distance. Variation over time is shown for each sample source. The significance of time variation was determined by a PERMANOVA test (adonis2 function of R packages vegan, 10000 permutations).

9 Stochasticity

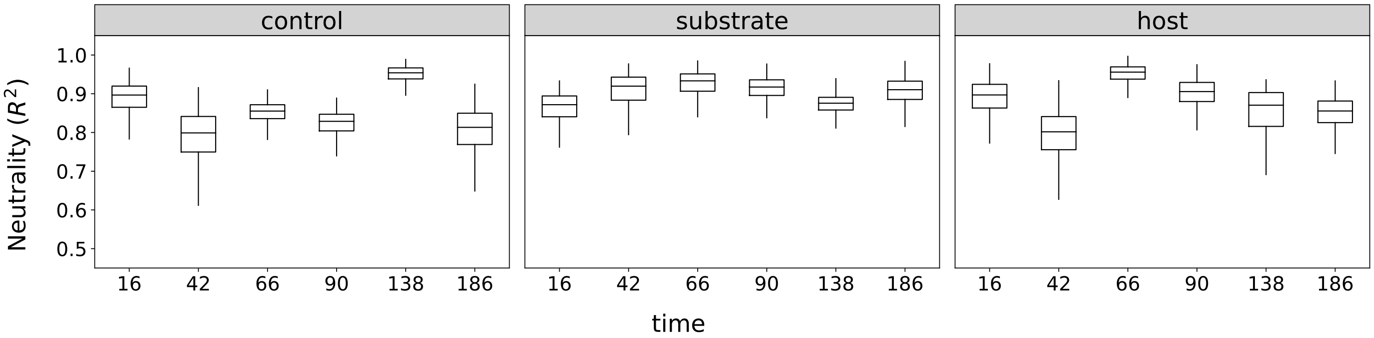

Figure S10: Goodness of fit of samples abundances to the neutral model. All samples sources are shown over time. High numbers of R^2 indicate that abundances match the neutral expectation.

10 Functional abundances

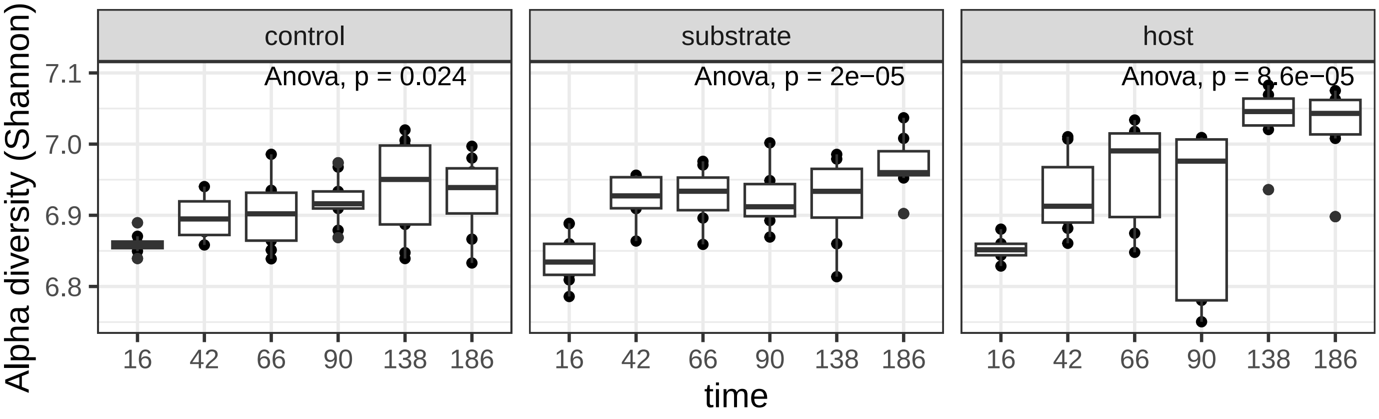

Figure S11: Shannon α-diversity of predicted functions. Predicted functions were paired with species abundance data, and diversity analysis was performed on functional abundances similar to diversity analysis of species abundances.

Table S11: Enrichment analysis of metabolic subsystems. We performed an over-representation analysis of differentially abundant metabolic pathways. The table shows significantly enriched subsystems.

Comparison	Subsystem	GeneRatio	BgRatio	pvalue	p.adjust	qvalue	Count
host vs. substrate	Secondary metabolite degradation	26/209	55/1014	0.0000	0.0001	0.0001	26
host vs. substrate	Carboxylate degradation	22/209	60/1014	0.0023	0.0249	0.0226	22
substrate vs. host	Aromatic compounds degradation	20/100	80/1014	0.0000	0.0007	0.0006	20
control vs. host	Aromatic compounds degradation	19/92	80/1014	0.0000	0.0007	0.0006	19

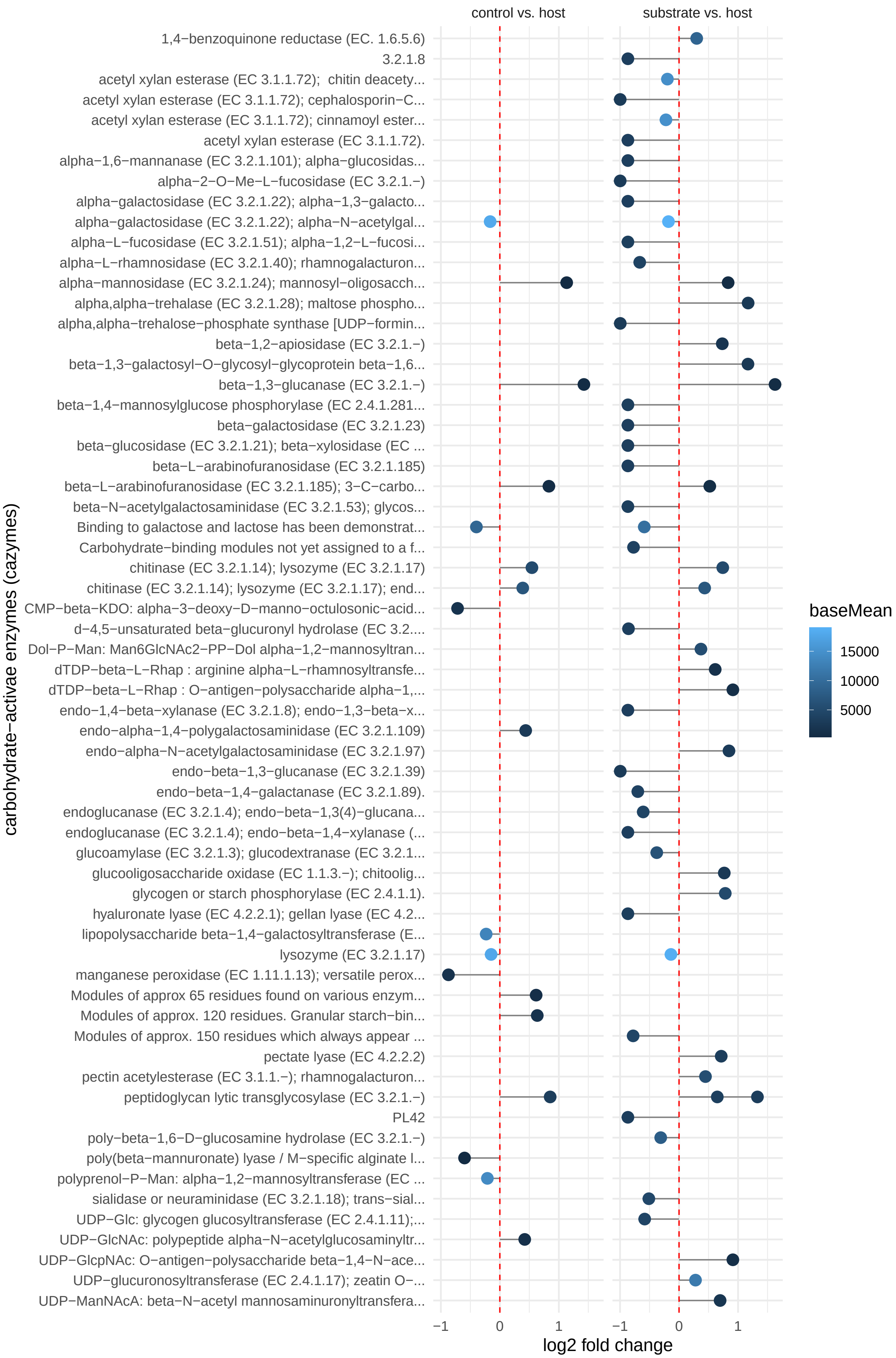

Figure S12: Enriched carbohydrate-active enzymes. Differential abundance analysis was used to compare the abundances of samples sources.

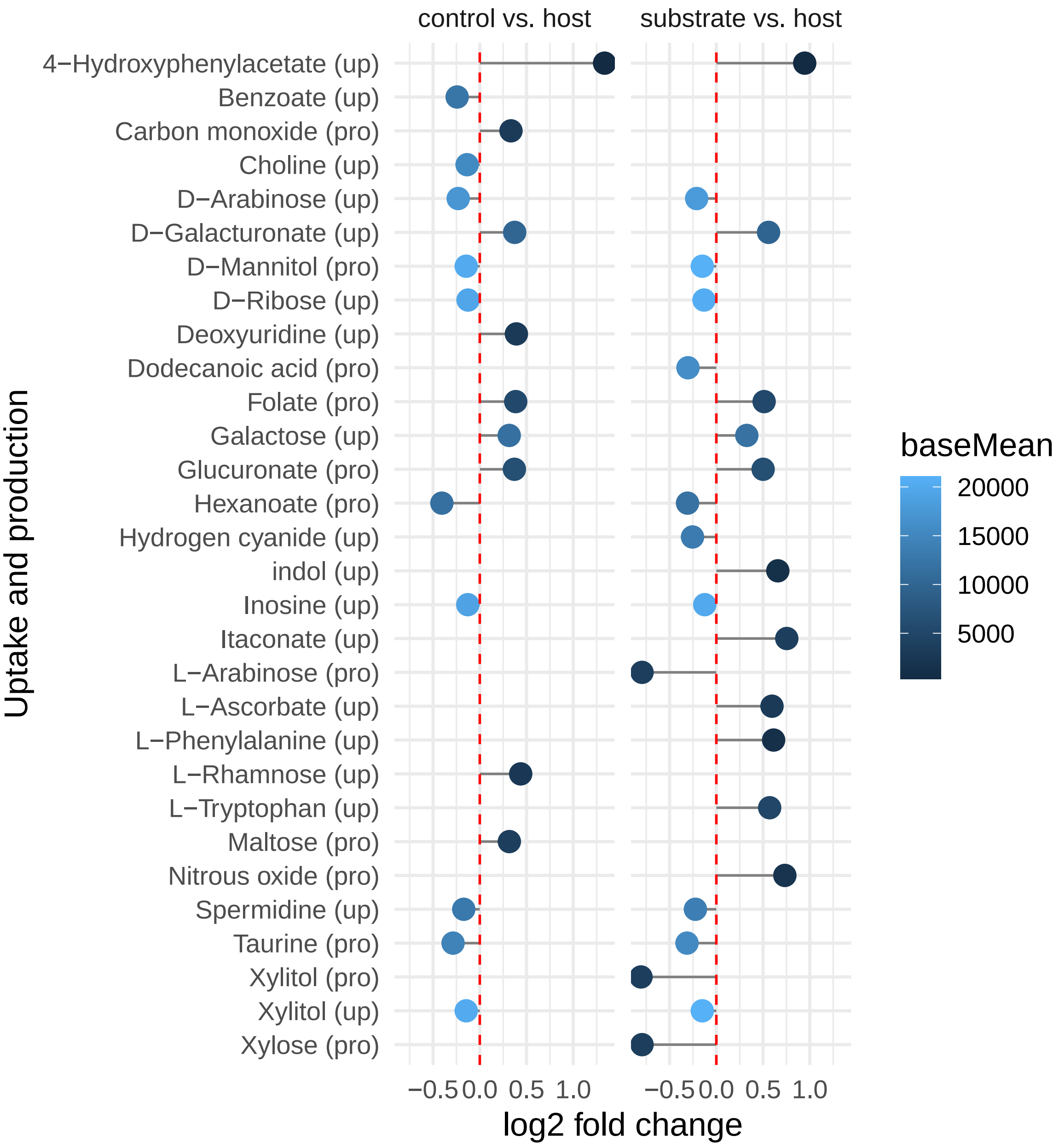

Figure S13: Enriched carbon/electron source (up) and metabolic by-products (pro) predicted from metabolic models using gapseq. Differential abundance analysis was used to compare the abundances of samples sources.

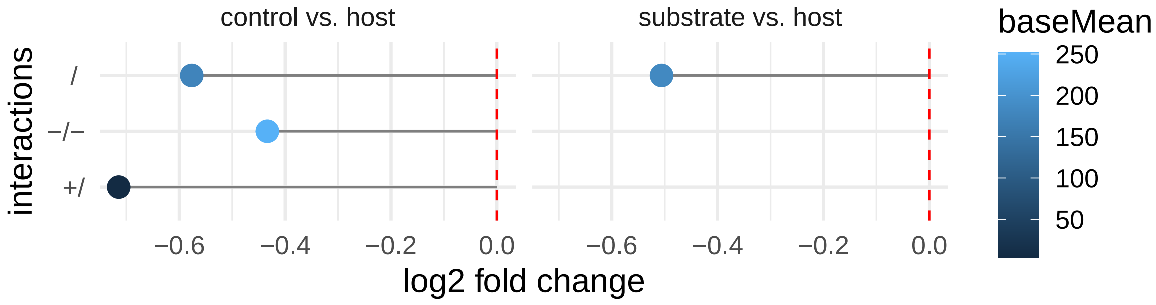

Figure S14: Enriched pair-wise interactions inferred from metabolic models by single vs. co-growth simulations and growth rate comparison. "/" indicates neutral interactions. Differential abundance analysis was used to compare the abundance of samples sources.

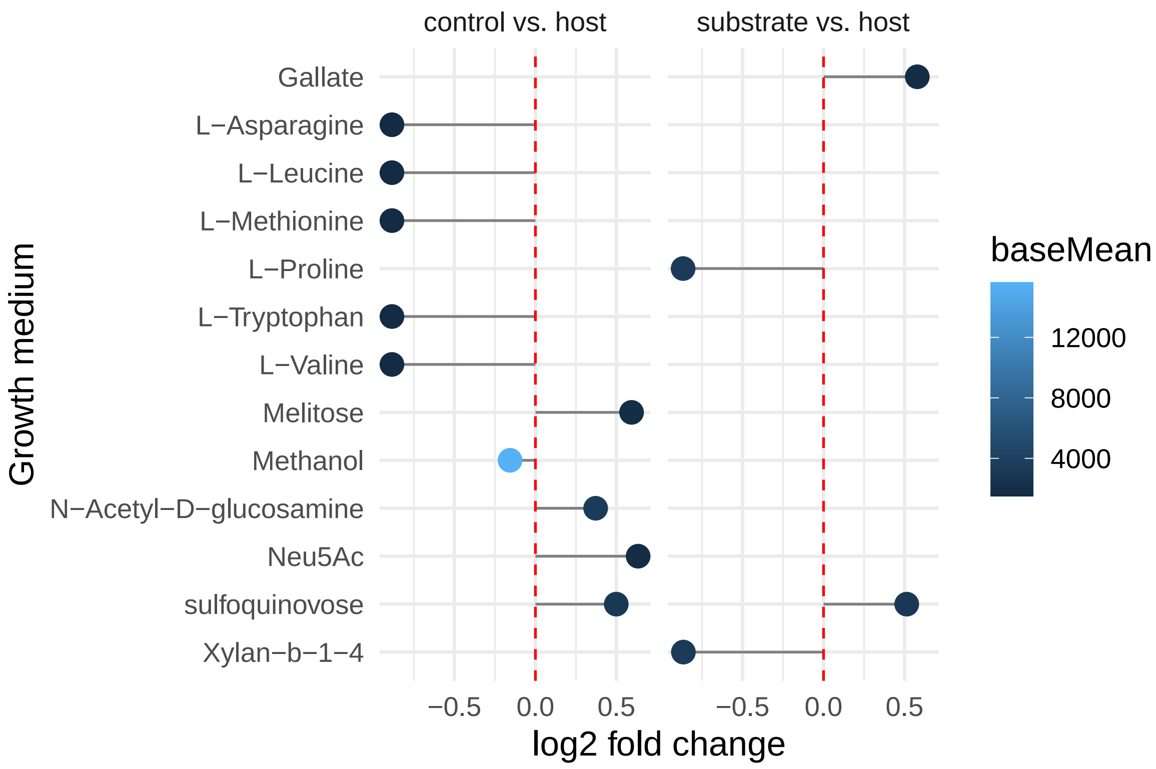

Figure S15: Enriched growth medium compounds predicted from metabolic models by gapseq. Abbreviation: N-acetylneuraminate (Neu5Ac). Differential abundance analysis was used to compare the abundance of samples sources.

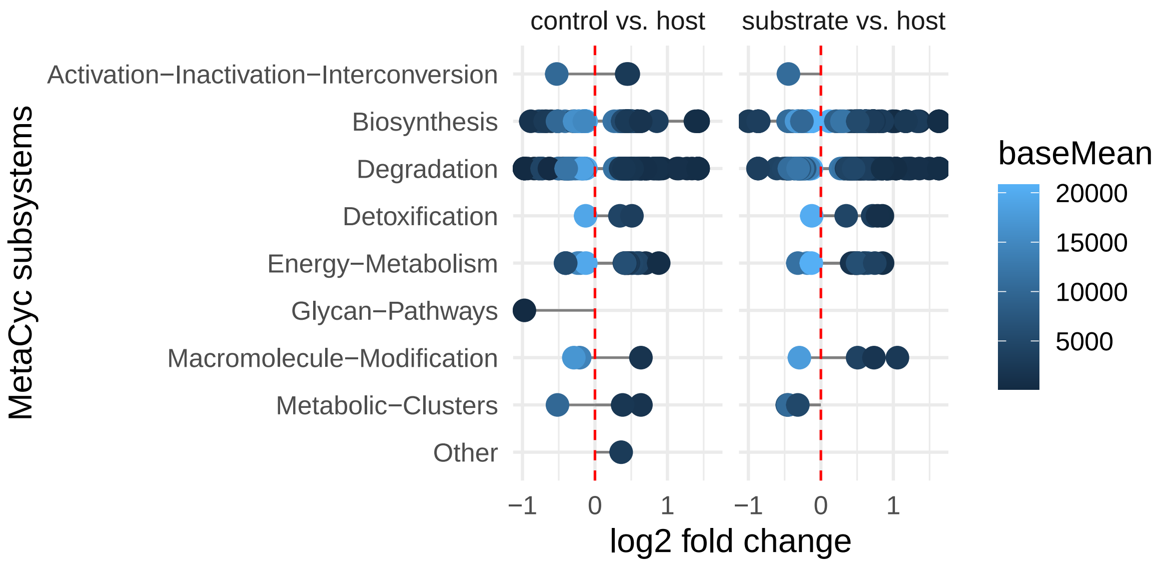

Figure S16: Enriched subsystem for MetaCyc for pathways predicted by gapseq. Differential abundance analysis was used to compare the abundance of samples sources.

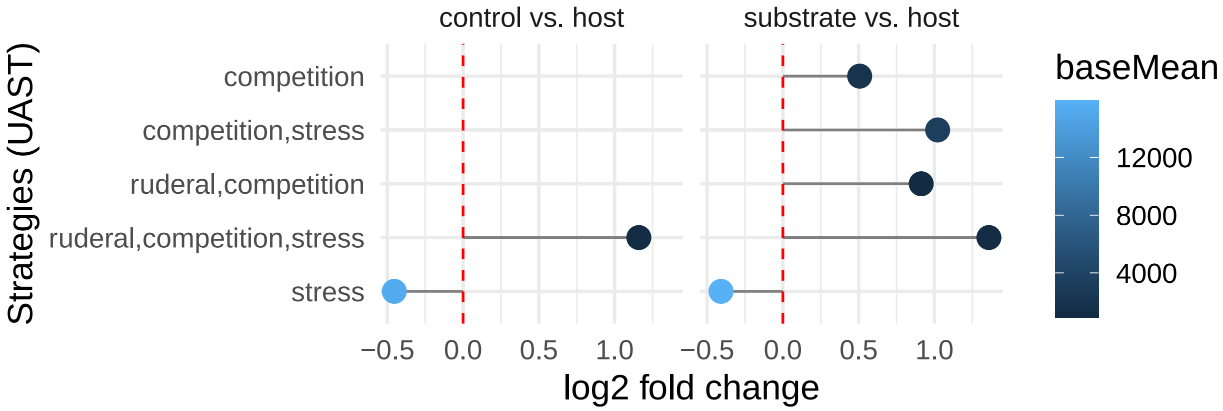

Figure S17: Enriched adaptive strategies and related traits as defined in the universal adaptive strategies (UAST) framework and as applied in a former study [39]. Differential abundance analysis was used to compare the abundance of samples sources. stress: stress-tolerating strategy.

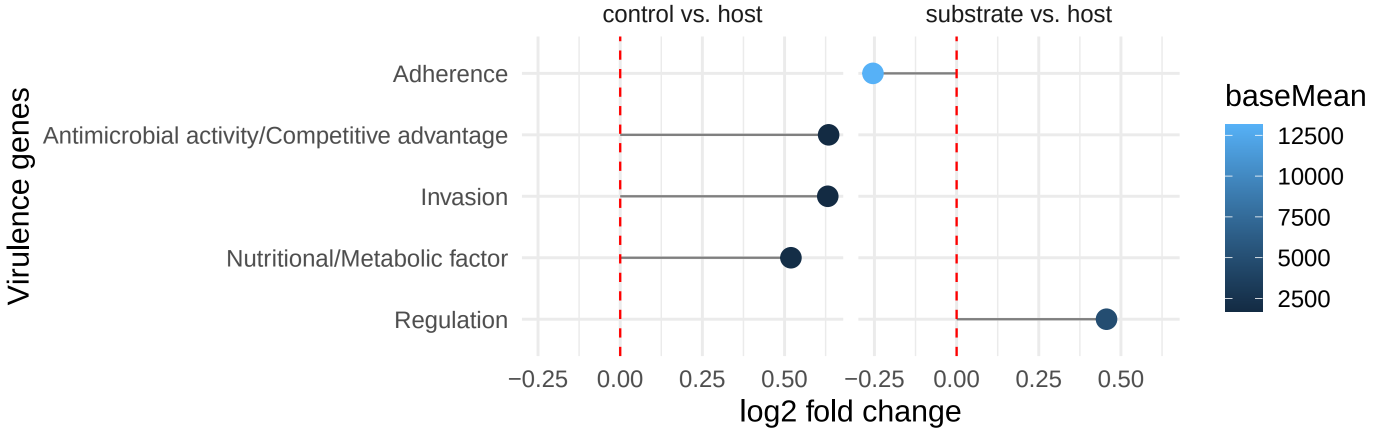

Figure S18: Enriched virulence factors predicted by abricate. Differential abundance analysis was used to compare the abundance of samples sources.

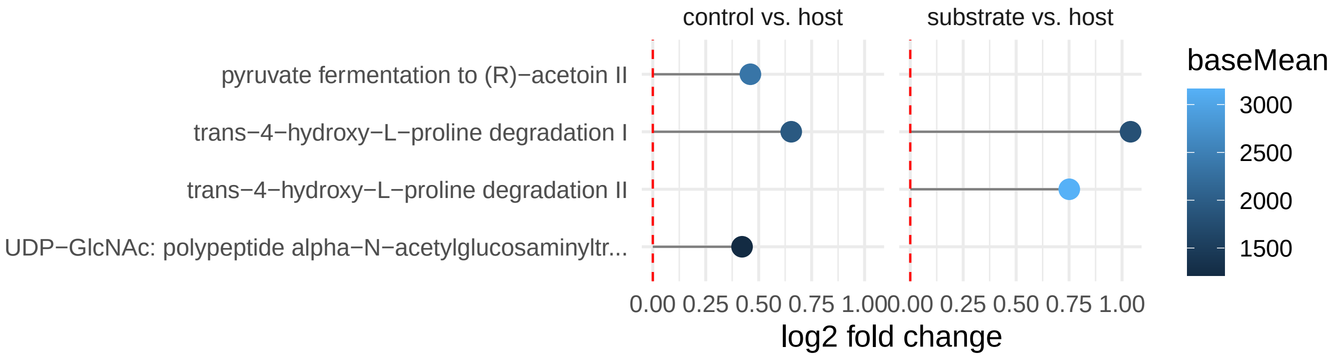

Figure S19: Enriched MetaCyc pathways for hydroxy-proline degradation and acetoin fermentation that we identified previously to be associated with worm fitness and bacterial load [39]. Differential abundance analysis was used to compare the abundance of samples sources.

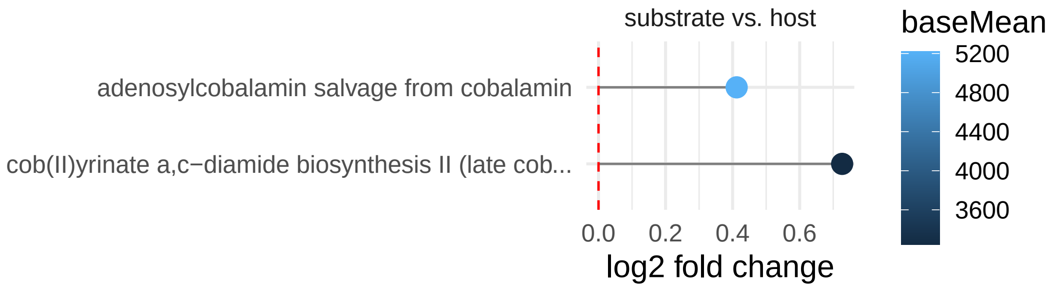

Figure S20: Enriched MetaCyc pathways involving vitamin B12. Differential abundance analysis was used to compare the abundance of samples sources.

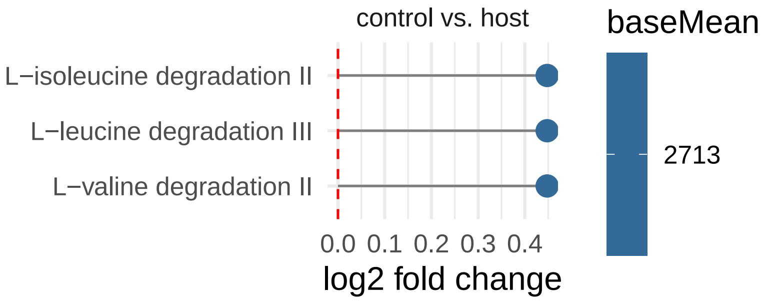

Figure S21: Enriched MetaCyc pathways relevant for branched-chain amino acids metabolism. Differential abundance analysis was used to compare the abundance of samples sources.

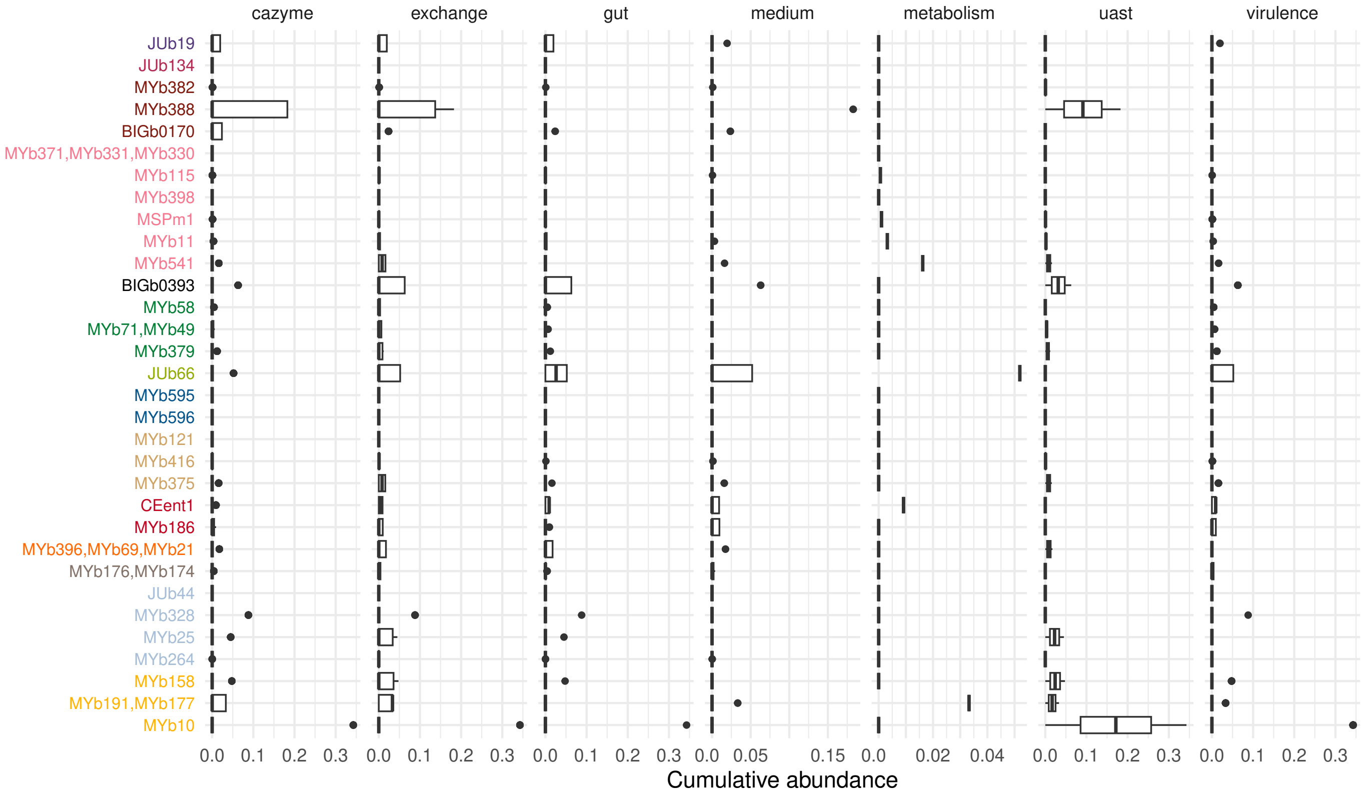

Figure S22: Contribution of microbial species to predicted functions. The attribution of individual taxa to predicted functions was determined by summing up the relative abundances for all species predicted to have the respective function.

11 Software

Table S12: Programs including the version numbers and the used parameter are listed. Detailed background on the specific applications of the programs can be found in the Materials and Methods section.

Program	Version	Parameter
abricate	0.8	--db vfdb
dbcan	3.0.2	prok -c cluster
gutSMASH	1.0.0-1555cd7	--genefinding-tool prodigal --cb-knownclusters --cb-general --enable-genefunctions
R	4.1	
tNST (R package NST)	3.1.10	rand=1000
nst.boot (R package NST)	3.1.10	rand=1000
BLAST	2.10.1+	perc_identity 99 -qcov_hsp_perc 95
BLAST (blobtools)	2.10.1+	-max_target_seqs 1 -max_hsps 1 -evalue 1e-25
PRINSEQ-lite	0.20.4	-min_len 20 -ns_max_n 8 -min_qual_mean 15 -trim_qual_left 12 -trim_qual_right 12
MaSuRCA	3.4.2	USE_LINKING_MATES = 1, LIMIT_JUMP_COVERAGE = 60, A_PARAMETERS = cgwErrorRate = 0.25, JF_SIZE = 150000000
gutSMASH	1.0.0-1555cd7	--genefinding-tool prodigal --cb-knownclusters --enable-genefunctions
BacArena	1.8.2	Bac: setAllExInf=TRUE, cellweight_sd=0, limit_growth=F
		Arena: time=3, sec_obj="mtf"
fastq_illumina_filter	0.1	--keep N -vv
SPAdes	v3.14.1	--careful --only-assembler
GtdbTk	2.3.2	identify, align, classify (default settings)
iqtree	2.2.3	-s gtdbtk.bac120.user_msa.fasta.gz -bb 1000 -T 16

References

[1] Anton Bankevich et al. "SPAdes: a new genome assembly algorithm and its applications to single-cell sequencing." eng. In: *J Comput Biol* 19.5 (May 2012), pp. 455–477. URL: http://dx.doi.org/10.1089/cmb.2012.0021.

[2] Eugen Bauer et al. "BacArena: Individual-based metabolic modeling of heterogeneous microbes in complex communities". In: *PLOS Computational Biology* 13.5 (2017), pp. 1–22. URL: https://doi.org/10.1371/journal.pcbi.1005544.

[3] Brian Bushnell. *BBMap short read aligner, and other bioinformatic tools*. sourceforge. 2020. URL: https://sourceforge.net/projects/bbmap/.

[4] Benjamin J Callahan et al. "DADA2: High-resolution sample inference from Illumina amplicon data". en. In: *Nat. Methods* 13.7 (July 2016), pp. 581–583. URL: https://doi.org/10.1038/nmeth.3869.

[5] Christiam Camacho et al. "BLAST+: architecture and applications." In: *BMC Bioinformatics* 10 (2009), p. 421. URL: https://doi.org/10.1186/1471-2105-10-421.

[6] Alessandra Carattoli et al. "In silico detection and typing of plasmids using PlasmidFinder and plasmid multilocus sequence typing". en. In: *Antimicrob. Agents Chemother.* 58.7 (July 2014), pp. 3895–3903. URL: https://doi.org/10.1128/aac.02412-14.

[7] Lihong Chen et al. "VFDB 2016: hierarchical and refined dataset for big data analysis—10 years on". In: *Nucleic acids research* 44.D1 (2015), pp. D694–D697. URL: https://doi.org/10.1093/nar/gkv1239.

[8] Philipp Dirksen et al. "CeMbio - The Caenorhabditis elegans Microbiome Resource". In: *G3: Genes, Genomes, Genetics* (July 2020), g3.401309.2020. URL: https://doi.org/10.1534/g3.120.401309.

[9] Philipp Dirksen et al. "The native microbiome of the nematode Caenorhabditis elegans: gateway to a new host-microbiome model". In: *BMC Biology* 14.1 (May 2016). URL: https://doi.org/10.1186/s12915-016-0258-1.

[10] Enrique Doster et al. "MEGARes 2.0: a database for classification of antimicrobial drug, biocide and metal resistance determinants in metagenomic sequence data". en. In: *Nucleic Acids Res.* 48.D1 (Jan. 2020), pp. D561–D569. URL: https://doi.org/10.1093/nar/gkz1010.

[11] Alexander P. Douglass et al. "Coverage-Versus-Length Plots, a Simple Quality Control Step for de Novo Yeast Genome Sequence Assemblies". In: *G3: Genes, Genomes, Genetics* 9.3 (2019), pp. 879–887. eprint: https://www.g3journal.org/content/9/3/879.full.pdf. URL: https://www.g3journal.org/content/9/3/879.

[12] Zachary S L Foster, Thomas J Sharpton, and Niklaus J Grünwald. "Metacoder: An R package for visualization and manipulation of community taxonomic diversity data". en. In: *PLoS Comput. Biol.* 13.2 (Feb. 2017), e1005404. URL: https://doi.org/10.1371/journal.pcbi.1005404.

[13] Bastian V H Hornung, Romy D Zwittink, and Ed J Kuijper. "Issues and current standards of controls in microbiome research". en. In: *FEMS Microbiol. Ecol.* 95.5 (May 2019). URL: https://doi.org/10.1093/femsec/fiz045.

[14] Julia Johnke, Philipp Dirksen, and Hinrich Schulenburg. "Community assembly of the native C. elegans microbiome is influenced by time, substrate and individual bacterial taxa." In: *Environ Microbiol* 22 (2020), pp. 1265–1279. URL: https://doi.org/10.1111/1462-2920.14932.

[15] DR Laetsch and ML Blaxter. "BlobTools: Interrogation of genome assemblies". In: *F1000Research* 6.1287 (2017). URL: https://doi.org/10.12688/f1000research.12232.1.

[16] Heng Li. "Aligning sequence reads, clone sequences and assembly contigs with BWA-MEM". In: (2013). URL: https://doi.org/10.48550/arXiv.1303.3997.

[17] Heng Li et al. “The sequence alignment/map format and SAMtools”. In: *Bioinformatics* 25.16 (2009), pp. 2078–2079. URL: https://doi.org/10.1093/bioinformatics/btp352.

[18] Michael I. Love, Wolfgang Huber, and Simon Anders. “Moderated estimation of fold change and dispersion for RNA-seq data with DESeq2”. In: *Genome Biology* 15.12 (Dec. 2014), p. 550. URL: https://doi.org/10.1186/s13059-014-0550-8.

[19] Paul J McMurdie and Susan Holmes. “phyloseq: an R package for reproducible interactive analysis and graphics of microbiome census data”. en. In: *PLoS One* 8.4 (Apr. 2013), e61217. URL: https://doi.org/10.1371/journal.pone.0061217.

[20] Alla Mikheenko et al. “Versatile genome assembly evaluation with QUAST-LG.” In: *Bioinformatics* 34 (2018), pp. i142–i150. URL: https://doi.org/10.1093/bioinformatics/bty266.

[21] Daliang Ning et al. “A general framework for quantitatively assessing ecological stochasticity”. In: *Proceedings of the National Academy of Sciences* 116.34 (Aug. 2019), pp. 16892–16898. URL: https://doi.org/10.1073/pnas.1904623116.

[22] Andrei Papkou et al. “The genomic basis of Red Queen dynamics during rapid reciprocal host–pathogen coevolution”. In: *Proceedings of the National Academy of Sciences* 116.3 (2019), pp. 923–928. URL: https://doi.org/10.1073/pnas.1810402116.

[23] Victòria Pascal Andreu et al. “The gutSMASH web server: automated identification of primary metabolic gene clusters from the gut microbiota”. In: *Nucleic Acids Research* 49.W1 (May 2021), W263–W270. eprint: https://academic.oup.com/nar/article-pdf/49/W1/W263/38842391/gkab353.pdf. URL: https://doi.org/10.1093/nar/gkab353.

[24] Carola Petersen et al. “Host and microbiome jointly contribute to environmental adaptation”. In: *The ISME Journal* (Sept. 2023).

[25] Simone Picelli et al. “Tn5 transposase and tagmentation procedures for massively scaled sequencing projects”. In: *Genome research* 24.12 (2014), pp. 2033–2040. URL: https://doi.org/10.1101/gr.177881.114.

[26] Robert Schmieder and Robert Edwards. “Quality control and preprocessing of metagenomic datasets”. In: *Bioinformatics* 27.6 (Jan. 2011), pp. 863–864. eprint: https://academic.oup.com/bioinformatics/article-pdf/27/6/863/646767/btr026.pdf. URL: https://doi.org/10.1093/bioinformatics/btr026.

[27] Thorsten Seemann. *ABRicate: Mass screening of contigs for antimicrobial and virulence genes.* github. 2020. URL: https://github.com/tseemann/abricate.

[28] Torsten Seemann. *Shovill: Assemble bacterial isolate genomes from Illumina paired-end reads.* github. 2020. URL: https://github.com/tseemann/shovill.

[29] Michael Sieber et al. “Neutrality in the Metaorganism.” In: *PLoS Biol.* 17 (2019), e3000298. URL: https://doi.org/10.1371/journal.pbio.3000298.

[30] William T Sloan et al. “Quantifying the roles of immigration and chance in shaping prokaryote community structure”. In: *Environmental Microbiology* 8.4 (2006), pp. 732–740. URL: https://doi.org/10.1111/j.1462-2920.2005.00956.x.

[31] Alexandre Souvorov, Richa Agarwala, and David J Lipman. “SKESA: strategic k-mer extension for scrupulous assemblies”. en. In: *Genome Biol.* 19.1 (Oct. 2018), p. 153. URL: https://doi.org/10.1186/s13059-018-1540-z.

[32] Theresa Stiernagle. “Maintenance of C. elegans”. In: *WormBook* (2006).

[33] Daniel Straub et al. “Interpretations of environmental microbial community studies are biased by the selected 16S rRNA (gene) amplicon sequencing pipeline”. en. In: *Front. Microbiol.* 11 (Oct. 2020), p. 550420. URL: https://doi.org/10.3389/fmicb.2020.550420.

[34] Ole Tange. *Gnu Parallel 2018*. en. Zenodo, 2018. URL: https://zenodo.org/record/1146014.

[35] Ryan R Wick et al. "Unicycler: Resolving bacterial genome assemblies from short and long sequencing reads." In: *PLoS Comput. Biol.* 13 (2017), e1005595. URL: https://doi.org/10.1371/journal.pcbi.1005595.

[36] Ea Zankari et al. "Identification of acquired antimicrobial resistance genes". en. In: *J. Antimicrob. Chemother.* 67.11 (Nov. 2012), pp. 2640–2644. URL: https://doi.org/10.1093/jac/dks261.

[37] Han Zhang et al. "dbCAN2: a meta server for automated carbohydrate-active enzyme annotation". In: *Nucleic Acids Research* 46.W1 (2018), W95–W101. URL: http://dx.doi.org/10.1093/nar/gky418.

[38] A. V. Zimin et al. "The MaSuRCA genome assembler". In: *Bioinformatics* 29.21 (Nov. 2013), pp. 2669–2677. URL: http://dx.doi.org/10.1093/bioinformatics/btt476.

[39] Johannes Zimmermann* et al. "The functional repertoire contained within the native microbiota of the model nematode *Caenorhabditis elegans*." In: *ISME Journal* 14.1 (2020), pp. 26–38. URL: https://doi.org/10.1038/s41396-019-0504-y.
